# Schizophrenia as a Network Disease: Disruption of Emergent Brain Function in Patients with Auditory Hallucinations

**DOI:** 10.1371/journal.pone.0050625

**Published:** 2013-01-21

**Authors:** Irina Rish, Guillermo Cecchi, Benjamin Thyreau, Bertrand Thirion, Marion Plaze, Marie Laure Paillere-Martinot, Catherine Martelli, Jean-Luc Martinot, Jean-Baptiste Poline

**Affiliations:** 1 Computational Biology Center, IBM T. J. Watson Research Center, Yorktown Heights, New York, United States of America; 2 Neurospin, CEA, Saclay, France; 3 EPI Parietal, INRIA-Saclay-Île-de-France, Saclay, France; 4 INSERM-CEA-Univ. Paris Sud, Research Unit U.797, Neuroimaging & Psychiatry, SHFJ & Neurospin, Orsay, France; 5 AP-HP, Adolescent Psychopathology and Medicine Department, Maison de Solenn, Cochin Hospital, University Paris Descartes, Paris, France; 6 Departement de Psychiatrie et d’Addictologie, Centre Hospitalier Paul Brousse, Villejuif, France; University of Buenos Aires, Argentina

## Abstract

Schizophrenia is a psychiatric disorder that has eluded characterization in terms of local abnormalities of brain activity, and is hypothesized to affect the collective, “emergent” working of the brain. Indeed, several recent publications have demonstrated that functional networks in the schizophrenic brain display disrupted topological properties. However, is it possible to explain such abnormalities just by alteration of local activation patterns? This work suggests a negative answer to this question, demonstrating that significant disruption of the topological and spatial structure of functional MRI networks in schizophrenia (a) cannot be explained by a disruption to area-based task-dependent responses, i.e. indeed relates to the emergent properties, (b) is global in nature, affecting most dramatically long-distance correlations, and (c) can be leveraged to achieve high classification accuracy (93%) when discriminating between schizophrenic vs control subjects based just on a single fMRI experiment using a simple auditory task. While the prior work on schizophrenia networks has been primarily focused on discovering statistically significant differences in network properties, this work extends the prior art by exploring the generalization (prediction) ability of network models for schizophrenia, which is not necessarily captured by such significance tests.

## Introduction

The concept of network disease, i.e. a dysfunction that affects the coordinated activity of a biological system, is receiving increased attention across all fields of biology and medicine. Though incomplete, current knowledge of protein-protein and gene-gene interaction networks provides a solid basis for assigning functional value to topological features such as connectivity, centrality, and so on [1], [2]. In neuroscience, the complexity of neural architecture and physiology precludes a similar detailed analysis. While Diffusion Tensor Imaging can reveal structural abnormalities associated with disease in large fiber tracts [3], [4], it is not immediately evident how these may affect the brain function.

Schizophrenia is in this sense a paradigmatic case. Unlike some other brain disorders (e.g., stroke or Parkinson’s disease), schizophrenia appears to be “delocalized”, i.e. difficult to attribute to a dysfunction of some particular brain areas. The failure to identify specific areas, as well as the controversy over which localized mechanisms are responsible for the symptoms associated with schizophrenia, have led us amongst many others (see, for example, [5]–[7]) to hypothesize that this disease may be better understood as a disruption of the emergent, collective properties of normal brain states. These emergent properties can be better captured by functional networks, based on inter-voxel correlation strength, as opposed to individual *voxel activations* localized in specific, task-dependent areas.

To test the hypothesis that schizophrenia, or any other psychiatric dysfunction, for that matter, is a network disease, we need first to clarify how to distinguish it from a *non-network disease*. In the first place, a network disease must have a measurable impact on one or several topological graph features of the associated functional brain networks in affected individuals, in comparison with control subjects. This has been the subject of several recent studies, reviewed later in the Discussion section, and needs no further discussion. However, while some disruption of topological features appears to be a *necessary* condition for a disease to be called a network dysfunction, it is not yet a *sufficient* one. Trivially, the topology of any sufficiently connected and structured graph can be significantly altered by the removal of a few nodes; this alteration would affect the network properties but *its cause would still be localized*. (As several studies seem to indicate, the brain behaves globally like a small-world and scale-free network [8], and as such it is prone to large disruptions if its hubs are affected [9]).

On the other hand, disruptions of network links that *cannot be explained just by local abnormalities* (e.g., when nodes remain intact) better fits an intuitive notion of a network disease. A distinction between node disruptions versus connectivity issues can be also linked to different biological phenomena behind such abnormalities. For example, while stroke is associated with neuronal death in specific areas, and thus can be viewed primarily as a local disfunction, schizophrenia is known to be associated with abnormal functioning of neurotransmitters, such as dopamine and glutamate, that can dramatically change the *functional* connectivity of a brain, even though underlying anatomical/structural elements may still remain intact (e.g., temporary drug-induced psychosis in healthy individuals, based on altering neurotransmitters, closely mimics positive symptoms of schizophrenia).

**Figure 1:**
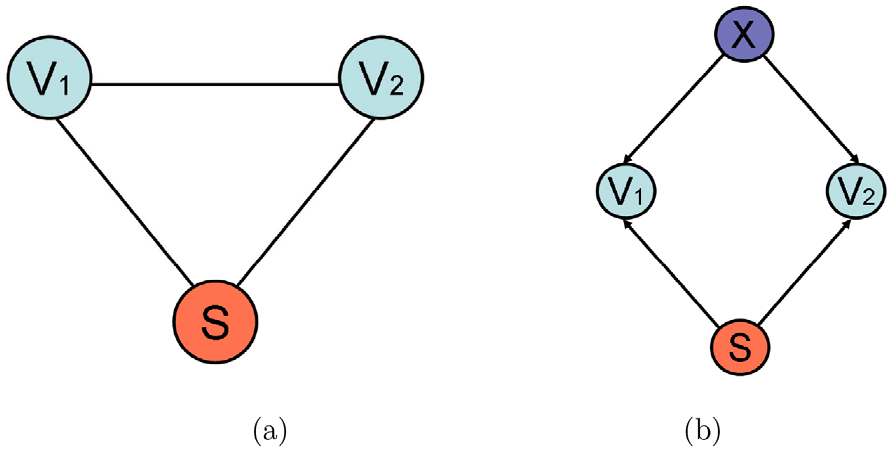
Graphical models of voxel interactions. Simple probabilistic graphical models capturing interactions among voxel-level BOLD signals and observed stimulus: (a) Markov network (undirected graph) over a pair of voxels and the task; (b) Bayesian network (directed graph) that includes an unobserved variable capturing other brain processes, besides the response to the observed stimulus, that can affect the BOLD signals. Note that directed links in Baysian networks are often (though not always) used to depict potential causal dependencies among the variables.

The following probabilistic model illustrates a situation where functional network connectivity disruptions occur independently from local (univariate) voxel activations. Let 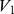 and 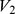 denote BOLD signals recorded by fMRI for a given pair of voxels, and let *S* represent a task, or a stimulus (such as, for example, an auditory task described later in this paper). Figure 1a depicts a simple Markov network encoding the structure of dependencies among these three variables. (A Markov network [10] is an undirected probabilistic graphical model, i.e. a graph associated with a joint probability distribution over the nodes, where a missing edge between a pair of variables encodes their conditional independence given the rest of the variables in the network.) We now assume there are two groups of subjects, e.g. schizophrenics and controls; for each group 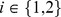, we can write the corresponding joint probability distribution in a factorized form as 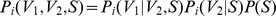, where we also assume that same task or stimulus *S* is applied to both groups of subjects, so that 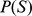 is fixed. Next, we assume that the stimulus has same effect on the voxel activity across the groups, i.e. there are no group-dependent local changes; more formally, we assume that 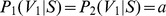 and 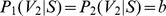. However, *even though each marginal distribution, i.e. 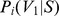 and 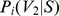, does not change across the subject group i, the conditional distribution 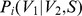, describing interactions among the pair of voxels, can vary across the groups*, since the constraint 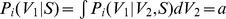 does not uniquely determine 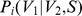. This illustrates how the voxel connectivity (described by their conditional distribution) can be altered across the two groups of subjects, without any change in the individual behavior of those voxels (described by their marginals).

Note that standard GLM approach focuses on univariate voxel *activations*, which are essentially just pairwise correlations, denoted herein as 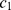 and 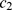, between the stimulus and each voxel’s signal 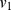 and 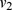, respectively. However, even if the values of 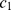 and 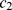 are *exactly* the same (or, more realistically, their difference is not statistically significant between the two groups of subjects, e.g. controls vs. schizophrenic patients), the pairwise correlation 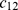 between the two voxels can still vary, unless one of the voxels is perfectly correlated with the stimulus (i.e., either 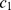 and 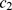 is exactly one, an extremely unlikely situation in practice).

A simple intuitive explanation behind varying inter-voxel functional connectivity in presence of fixed univariate stimulus-based activations is that there are multiple ongoing brain processes, besides the observed stimulus, that also affect the BOLD signal, and can be summarized as a hidden (unobserved) variable. Figure 1b depicts a *directed* probabilistic graphical model, or Bayesian network, demonstrating such situation. A naturally arising hypothesis is that some of those processes can be disrupted in schizophrenic patients, leading to disturbed interactions among voxels, even if the task-based voxel activations might be similar to those of the controls.

We can gain further insight by analyzing more mechanistic models of brain activity. In particular, let us first consider two approaches that have been utilized frequently as models of interacting neuronal ensembles: coupled non-linear relaxation oscillators, defined by their phases and frequency of oscillation [11], [12], and Ising systems of coupled spins subject to an inhomogeneous external field [13]. It is possible to show that for the case of coupled oscillators, varying the coupling strength over a wide range of values leads to dramatic changes in the correlation between the units, without significantly affecting the individual rates (as measured by the frequency of oscillation). Similarly, for a fixed external field, it can be shown that the mean magnetization remains constant as the spin-spin correlation changes, as a function of a varying coupling strength. Moreover, even if a *linear* system driven by a multi-dimensional Gaussian process is not properly inferred (for instance, by assuming that the process is homogeneous over the nodes, when it is not), one may confound a change in the mean activity of a node by a change in the connectivity of the system. The detailed calculations for these three models are presented in Material S1.

Thus, when analyzing a brain disorder associated with functional network abnormalities, one should first test the null-hypothesis assuming that the abnormalities can be fully explained by local disruptions; rejection of such null-hypothesis would provide a solid basis for classifying the observations as a truly network disorder. However, given the limited spatio-temporal resolution of current imaging techniques, a thorough analysis of this null hypothesis can be carried out only partially, and in the best cases requiring heavy computational resources or dramatic dimensionality reduction [14], [15]. We propose, however, an alternative approach suitable for the type of data provided by fMRI: if schizophrenia is a network disease, we would expect the multi-variate functional properties captured by topological graph features to *carry more population-specific information* than univariate and localized analysis approaches such as the General Linear Model. This is a sufficient condition, but in general not necessary; nevertheless, if satisfied, it is a strong indication that the dysfunction cannot be simply reduced to local functional disruptions. We would like to stress at this point that when network effects are discussed, this aspect is typically overlooked.

Finally, in order to quantify the notion of information carried by network as opposed to localized features, we consider necessary to complement hypothesis-testing with predictive modeling/classification statistical approaches. Various reasons justify the use of predictive modeling for brain imaging. In particular, the classification framework evaluates the *generalization* ability of models built using the features of interest, i.e. the ability to predict whether a *previously unseen* subject is schizophrenic or not, unlike standard statistical hypothesis testing that evaluates the differences between two groups of subjects (e.g., schizophrenic and control) on a *fixed* dataset. Moreover, predictive modeling is more robust to the presence of heavy-tailed feature distributions, which naturally arise in the topological analysis of complex networks [8].

Following the above rationale, in subsequent sections we will demonstrate that network features reveal highly statistically significant differences between the schizophrenic and control groups; moreover, statistically significant subsets of certain network features, such as *voxel degrees* (the number of voxel’s neighbors in a network), are quite stable over varying data subsets. In contrast, voxel activation show much weaker group differences as well as stability. Moreover, most of the network features, and especially pairwise voxel correlations (*edge weights*) and voxel degrees, allow for quite accurate classification, as opposed to voxel activation features: degree features achieve up to 86% classification accuracy (with 50% baseline) using Markov Random Field (MRF) classifier, and even more remarkable 93% accuracy is obtained by linear Support Vector Machines (SVM) using just a dozen of the most-discriminative correlation features. We will also show evidence that traditional approaches based on a direct comparison of the correlation at the level of relevant regions of interest (ROIs) or using a functional parcellation techniques [16], do not reveal any statistically significant differences between the groups. Indeed, a more data-driven approach that exploits properties of voxel-level networks appears to be necessary in order to achieve high discriminative power. The results presented in this paper unify and extend the approaches presented in our earlier work in [17], [18].

## Materials and Methods

We first describe the experimental paradigm and the groups of participating subjects, second the region of interest analysis, and then the network analysis and classification methods used to assess our capacity to predict which subject is schizophrenic.

### Ethics Statement

Ethical approval was obtained from the Paris-Pitié-Salpétrière ethics committee. Participants were fully informed of the requirements of the behavioral task and all demonstrated that they understood the aims and demands of the experiment. All subjects gave written informed consent. The subjects’ ability to consent was established by clinical interviews, which demonstrated that this ability was not compromised by the subjects mental condition.

### Experimental Paradigm and Data Acquisition

In our studies, we worked with a group of 15 schizophrenic subjects (9 women) fulfilling DSM-IV-R criteria for schizophrenia with daily auditory hallucinations for at least 3 months despite well-conducted treatment. Their mean ± S.D. age was 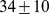 years (i.e., 22–49 years range), and the duration of illness was 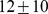 years (3–28 years range). All schizophrenic patients were treated with antipsychotic drugs (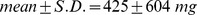) chlorpromazine equivalent/day [19].

Four subjects were discarded because of acquisition issues, leaving us with 11 subjects, that were approximately matched for gender and age by the control group of 11 healthy subjects. Originally, the dataset also included a group of alcoholic patients; however, in this paper, we focused primarily discriminating between the schizophrenic and normal groups; the results including the alcoholics group together with controls, and testing against the schizophrenic group, were quite similar to those presented here, and are included in Material S1.

**Figure 2:**
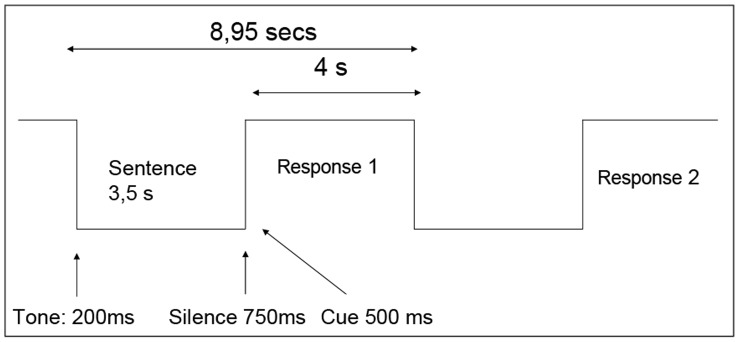
Experimental paradigm.

All subjects were submitted to the same experimental paradigm involving language (see Figure 2), which was similar to the one introduced in [20]. The task is based on auditory stimuli; subjects listen to emotionally neutral sentences either in native (French) or foreign language. Average length (3.5 sec mean) or pitch of both kinds of sentences is normalized. In order to catch attention of subjects, each trial begins with a short (200 ms) auditory tone, followed by the actual sentence. The subject’s attention is asserted through a simple validation task: after each played sentences, a short pause of 750 ms is followed by a 500 ms two-syllable auditory cue, which belongs to the previous sentence or not, to which the subject must answer to by yes (the cue is part of the previous sentence) or no with push-buttons, when the language of the sentence was his own. A full fMRI run contains 96 trials, with 32 sentences in French (native), 32 sentences in foreign languages, and 32 silence interval controls.

Data were acquired on a 1.5 T Signa (General Electric) Scanner at Service Hospitalier Frédéric Joliot, Orsay, France. For each subject, two fMRI runs are acquired (T2-weighted EPI), each of which consisted of 420-scans (from which the first 4 are discarded to eliminate T1 effect), with a repetition time (TR) of 2.0 second, for a total length of 14 minutes per run. Data were spatially realigned and warped into the MNI template and smoothed (FWHM of 5 mm) using SPM5 (www.fil.ucl.ac.uk); also, standard SPM5 motion correction was performed with the SPM5 realignment pre-processing. For each volume of the time-series, the process estimates a 6 degree-of-freedom movement relative to the first volume. These estimated parameters are combined to warping parameters (obtained by nonlinear deformation on an EPI template) to get the final, spatially normalized and realigned time-series. Finally, a universal mask was computed as the minimal intersection of thesholded EPI mean volumes across the entire dataset. This mask was then applied to all subjects.

Note that the schizophrenia patients studied here have been selected for their prominent, persistent, and pharmaco-resistant auditory hallucinations [20] which might have increased their clinical homogeneity, but they are not representative of all schizophrenia patients, only of a subgroup.

In summary, our dataset contained the total of 44 samples (there were two samples per subject, corresponding to the two runs), where each sample corresponds to a subject/run combination, and is associated with roughly 50,000 voxels × 420 TRs × 2 runs, i.e. more than 40,000,000 voxels/variables. In the subsequent sections, among other methods, we discuss feature-extraction approaches that reduce the dimensionality of the data prior to learning a predictive model.

### Methods

We explored two different data analysis approaches aimed at discovery of discriminative patterns: (1) model-driven approaches based on prior knowledge about the regions of interest (ROI) that are believed to be relevant to schizophrenia, or model-based functional clustering, and (2) data-driven approaches based on various features extracted from the fMRI data, such as standard activation maps and a set of topological features derived from functional networks.

### Model-Driven Approach using ROI

**Figure 3:**
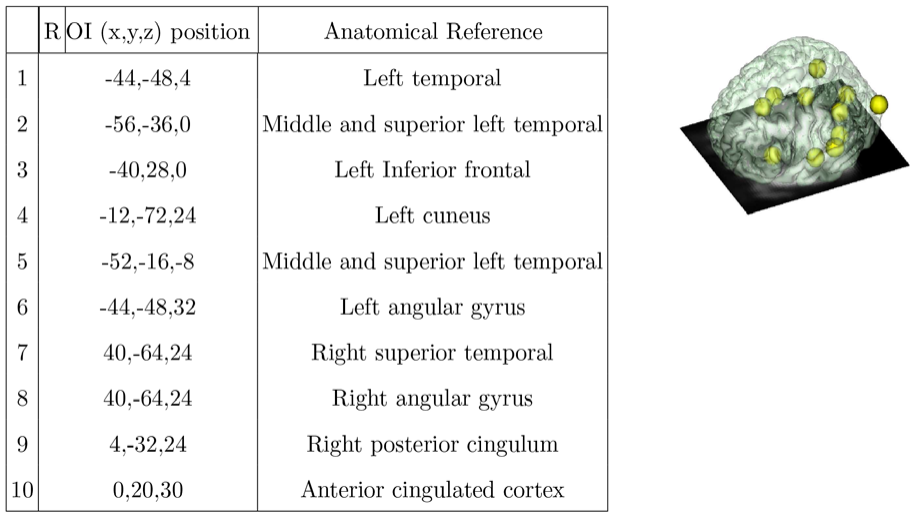
Locations of ROIs. Regions of Interest (ROI) and their location on a brain normalized to the MNI template (Talairach coordinate system). Note that the region outside the brain has been defined for testing purposes.

First, we decided to test whether the interactions between several known regions of interest (ROIs) would contain enough discriminative information about schizophrenic versus normal subjects. Ten regions of interests (ROI) were defined using previous literature [20] on schizophrenia and language studies, including inferior, middle and superior left temporal cortex, left inferior temporal cortex, left cuneus, left angular gyrus, right superior temporal, right angular gyrus, right posterior cingulum, and anterior cingular cortex (Figure 3). Each region was defined as a sphere of 12 mm diameter centered on the x,y,z coordinates of the corresponding ROI.

Because predefined regions of interest may be based on too much a priori knowledge and miss important areas, we also ran a more exploratory analysis. A second set of 600 ROI’s was defined automatically using a parcellation algorithm [16] that estimates, for each subject, a collection of regions based on functional signal similarity and position in the MNI space.

Time series were extracted as the spatial mean over each ROI, leading to 10 time series per subject for the predefined ROIs and 600 for the parcellation technique. Drifts were removed from the time series by removing low frequencies below 1/128 Hz using a cosine basis.

The connectivity measures were of two kinds. First, the correlation coefficients were computed between each pair of ROIs time series without taking into account the experimental paradigm. Next, we computed a psycho-physiological interaction (PPI), by contrasting the correlation coefficient weighted by experimental conditions (i.e. correlation weighted by the “Language French” condition versus correlation weighted by “Control” condition after convolution with a standard hemodynamic response function). Those connectivity measures were then tested for significance using standards non-parametric tests between groups (Wilcoxon signed-rank test) with corrected p-values for multiple comparisons.

### Data-driven Approach: Feature Extraction

#### Activation maps

To find out whether local task-dependent linear activations alone could possibly explain the differences between the schizophrenic and normal brains, we used as a baseline set of features based on the standard voxel *activation maps*, computed using General Linear Model (GLM). The GLM analysis described here is a standard component of the Statistical Parametric Mapping (SPM) toolkit. Given the time-series for stimulus 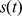 (e.g., s = 1 if the stimulus/event is present, and s = 0 otherwise), and the BOLD signal intensity time-series 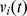 for voxel *i*, GLM is simply a linear regression 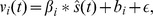 where 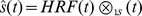 is the regressor corresponding to the stimulus convolved with the *hemodynamic response function (HRF)* in order to account for delay between the voxel activation and change in the BOLD signal, 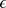 is noise, 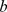 is the baseline (mean intensity) and 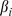 coefficient is the *amplitude* that serves as an *activation score* (note that 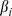 coefficient is simply the correlation between 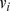 and 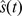 when both are normalized and centered prior to fitting the model). Given multiple trials, multiple estimates of 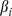 are obtained and a statistical test (e.g., t-test) is performed for the mean 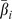 against the null-hypothesis that it comes from Gaussian noise distribution with zero mean and fixed noise 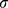 (the level of noise for BOLD signal is assumed to be known here).

In case of multiple stimuli, the GLM model uses a vector of regressors 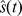 and obtains the vector of the corresponding coefficients 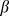. For example, in our studies, the following stimuli/events were considered: “FrenchNative”, “Foreign”, and “Silence”, together with several additional regressors, such as some low-frequencies trends and the movement parameters (additional 1-only column is added to account for the mean of the signal, as above - a standard step in linear regression with the unnormalized data). Once the GLM is fit, we focus on the 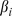 coefficients obtained for the above three stimuli, and the corresponding three activation maps. Next, we compute several *activation contrast maps* by subtracting some maps from the others (hoping that such differences, or contrasts, may provide additional information). The following activation contrast maps were computed: *activation contrast 1: “FrenchNative – Silence”*, *activation contrast 2: “FrenchNative – Foreign”*, *activation contrast 3: “Silence – FrenchNative”*, *activation contrast 4: Foreign - FrenchNative* (note that maps 2 and 4 are just negations of the maps 1 and 3, respectively), *activation contrast 5: “Foreign – Silence”*; also, the following three contrast maps are simply the difference of the corresponding 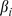 coefficient (activation) and the mean (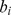): *activation contrast 6: “FrenchNative”*, *activation contrast 7: “Foreign”*, *activation contrast 8: “Silence”*. For each of those maps, *t*-values are computed at each voxel (with a null-hypothesis corresponding to zero-mean Gaussian). In the analysis presented in this paper, we use the resulting *t*-value maps, rather than just the “raw” activation maps (i.e., 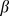 coefficient maps), and to simplify the terminology, just refer to them as “activation” or “activation contrast” maps.

The above activation contrast maps (that we will further refer to as simply activation maps) were computed for each subject and for each run. The activation values of each voxel were subsequently used as features in the classification task. We also computed a global feature, mean-activation (denoted *mean-t-val*), by taking the mean absolute value of the voxel’s t-statistics.

#### Network features

In order to continue investigating possible disruptions of global brain functioning associated with schizophrenia, we decided to explore lower-level (as compared to ROI-level) *functional* brain networks [8] constructed at the voxel level, as follows: (1) pair-wise Pearson correlation coefficients are computed among all pairs of time-series 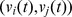 where 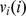 corresponds to the BOLD signal of *i*-th voxel; (2) an edge between a pair of voxels 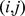 is included in the network if the correlation between 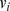 and 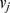 exceeds a specified threshold (herein, we used the same threshold of c(Pearson) = 0.7 for all voxel pairs; we tried a few other threshold levels, such as 0.8 and 0.9, and the results were similar; however, we did not perform an exhaustive evaluation of the full range of this parameter due to high computational cost of such experiment). For each subject, and each run, a separate functional network was constructed. Next, we measured a number of its *global* topological features, including:

1. *the mean degree*, i.e. the number of links for each node (corresponding to a voxel), averaged over the entire network;
2. *the mean geodesic distance*, i.e. the minimal number of links needed to reach any to from any other node, averaged over the entire network;
3. *the mean clustering coefficient*, i.e. the fraction of triangulations formed by a node with its first neighbors relative to all possible triangulations, averaged over the entire network;
4. *the giant component*, i.e. the size (number of nodes) of the largest connected subgraph in the network;
5. *the giant component ratio*, i.e. the ratio of the giant component size to the size of the network;
6. *the total number of links* in the network.

Besides global topological features, we also computed a series of *voxel-level* network features, based on topological properties of an individual voxel in functional network; the following types of features were used:

*(full) degree*: the value assigned to each voxel is the total number of links in the corresponding network node;
*long-distance degree*: the number of links making non-local connections (i.e., links between the given voxel and the voxels that are 5 or more voxels apart from it);
*inter-hemispheric degree*: only links reaching across the brain hemispheres are considered when computing each voxel’s degree;
*strength*: node strength is the sum of weights of links connected to the node. In our study, the full correlation matrix was used as a weighted adjacency matrix, where each pairwise correlation corresponds to the link weight; thus, for each voxel, its strength is the sum of its correlations with the other voxels;
*absolute strength*: same as above, but the link weights are replaced by their absolute values;
*positive strength*: same as node strength, but only positive link weights are considered;
*clustering coefficient* of a node is the fraction of triangles around a node, i.e. the fraction of node’s neighbors that are neighbors of each other; herein, we first computed a functional networks by applying a threshold of 0.7 to the absolute values of the pairwise correlations, and then used the resulting graph to compute the clustering coefficients for each node/voxel;
*local efficiency*: the local efficiency is the *global efficiency* computed on node neighborhoods, and is related to the clustering coefficient. the global efficiency is the average inverse shortest path length in the network, that is 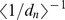, where 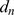 is the shortest path for node *n*, such that for disconnected nodes 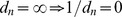;
*edge weights*: finally, we simply used as features a *randomly selected subset of* 200,000 *pairwise correlations* out of 53,000×53,000 entries of the correlation matrix (the location of pairs were randomly selected once, and then *same locations used to derive features for all subjects*); the rationale behind random sampling from the correlation matrix was to reduce the computational complexity of working with the full set of correlations, which would exceed 2800 million features. Nevertheless, subsequent feature ranking procedure was able to select a highly discriminative subset of correlation features, which would only improve if the feature ranking was allowed to continue running over the rest of the correlation matrix. (Note that we also tried other sets of randomly selected 200,000 voxels and obtained similar results to those presented in this paper. Clearly, the results may vary if we keep selecting other random sets of voxels that may not include the top most informative voxel pairs discovered in our analysis. However, the point of our analysis is to show that it is *possible* to find predictive features among pairwise correlations, and that our results demonstrate only a lower bound on a potentially even better predictive performance of correlation features.)

For each of the above feature types, except the edge weights, we call the corresponding feature sets “feature map”, since each voxel is associated with its own feature value, e.g. (full) degree maps, strength maps, and so on.

The set of global measures and spatial maps was utilized for further analysis of statistical significance of group differences, including t-test and several classification approaches, described below.

#### Spatial normalization

Note that, for each sample, we also computed *spatially normalized* activation and degree maps, dividing the corresponding maps by their maximal value taken over all voxels in the given map. As it turned out, normalization affected both statistical testing and classification results presented below. We mainly focus on normalized activation and degree maps (full, long-distance and inter-hemispheric), since they yield better classification results. In case of hypothesis testing, however, unnormalized (raw) activations maps, unlike the degree maps, happened to outperform their normalized counterparts, and thus both sets of results were presented.

### Classification Approaches

#### Classification tasks

We first focused on discriminating between the schizophrenic and normal subjects only, that resulted into well-balanced dataset containing 2×11 positive (schizophrenic) and 2×11 negative (healthy) samples (since there were two runs per each subject), with 50% baseline prediction accuracy. The results for the original dataset, including alcoholics together with controls into one category and discriminating them from schizophrenic subjects, were quite similar to those presented here, i.e. we were able to accurately separate schizophrenics from the alcoholic subjects merged with the controls; the results are included in Material S1.

#### Classifiers

First, standard off-the-shelf methods such as Gaussian Naive Bayes (GNB) and Support Vector Machines (SVM) were used in order to compare the discriminative power of different sets of features described above. We used standard SVM implementation with linear kernel and default parameters, available from the LIBSVM library. For GNB, we used our own MATLAB implementation.

Moreover, we decided to further investigate our hypothesis that interactions among voxels contain highly discriminative information, and compare those linear classifiers against probabilistic graphical models that explicitly model such interactions. Specifically, we learn a classifier based on a sparse Gaussian Markov Random Field (MRF) model [21], which leads to a convex problem with unique optimal solution, and can be solved efficiently; herein, we used the COVSEL procedure [21]. The weight on the 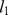-regularization penalty serves as a tuning parameter of the classifier, allowing to control the sparsity of the model, as described below.

#### Sparse Gaussian MRF classifier

Let 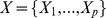 be a set of *p* random variables (e.g., voxels), and let 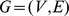 be an undirected graphical model (Markov Network, or MRF) representing conditional independence structure of the joint distribution 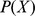. The set of vertices 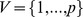 is in the one-to-one correspondence with the set *X*. The set of edges *E* contains the edge 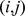 if and only if 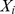 is conditionally dependent on 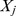 given all remaining variables; lack of edge between 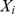 and 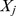 means that the two variables are conditionally independent given all remaining variables. Let 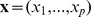 denote a random assignment to *X*. We will assume a multivariate Gaussian probability density 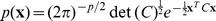, where 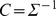 is the inverse covariance matrix (also called the *precision* matrix), and the variables are normalized to have zero mean. Let 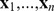 be a set of *n* i.i.d. samples from this distribution, and let 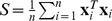 denote the empirical covariance matrix. Missing edges in the above graphical model correspond to zero entries in the inverse covariance matrix *C*, and thus the problem of learning the structure for the above probabilistic graphical model is equivalent to the problem of learning the zero-pattern of the inverse-covariance matrix. Note that the inverse of the *empirical* covariance matrix, even if it exists, does not typically contain exact zeros. Therefore, an explicit sparsity constraint is usually added to the estimation process. A popular approach is to use 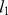-norm regularization that is known to promote sparse solutions, while still allowing (unlike non-convex 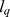-norm regularization with 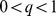) for efficient optimization. From the Bayesian point of view, this is equivalent to assuming that the parameters of the inverse covariance matrix 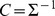 are independent random variables 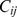 following the Laplace distributions 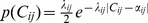 with zero *location parameters* (means) 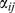 and equal *scale parameters* 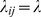. Then 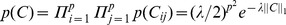, where 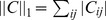 is the (vector) 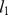-norm of *C*.

Assume a fixed parameter 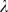, our objective is to find 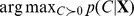, where 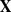 is the 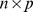 data matrix, or equivalently, since 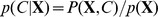 and 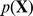 does not include *C*, to find 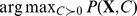, over positive definite matrices *C*. This yields the following optimization problem considered, for example, in [21]

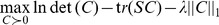
(1)where 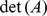 and 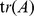 denote the determinant and the trace (the sum of the diagonal elements) of a matrix *A*, respectively, and S the empirical covariance of the data. For the classification task, we estimate on the training data the Gaussian conditional density 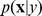 (i.e. the (inverse) covariance matrix parameter) for each class 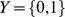 (schizophrenic vs control), and then choose the most-likely class label 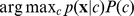 for each unlabeled test sample **x**.

#### Variable selection

Note that each sample is associated with roughly 50,000 voxels × 420 TRs × 2 runs, i.e. more than 40,000,000 voxels/variables. Thus, some kind of dimensionality reduction and/or feature extraction appears to be necessary prior to learning a predictive model. Extracting degree maps and activation maps reduced dimensionality by collapsing the data along the time dimension.

Moreover, we used variable selection as an additional preprocessing step before applying a particular classifier, in order to (1) further reduce the computational complexity of classification (especially for sparse MRF, which, unlike GNB and SVM, could not be directly applied to 50,000 variables), (2) reduce noise and (3) identify relatively small predictive subsets of voxels. We applied a simple filter-based approach, selecting a subset of top-ranked voxels, where the ranking criterion used p-values resulting from the paired t-test, with the null-hypothesis being that the voxel values corresponding to schizophrenic and non-schizophrenic subjects came from distributions with equal means. The variables were ranked in the ascending order of their p-values (lower p-values correspond to higher confidence in between-group differences), and classification results on top *k* voxels will be presented for a range of *k* values. Clearly, in order to avoid biased estimate of generalization error, variable selection was performed *separately* on each cross-validation training dataset; failure to do so, i.e. variable selection on the full dataset, would produce overly optimistic results with nearly-perfect accuracy (e.g., 95% accuracy using GNB on just 100 top t-test voxels).

#### Evaluation via cross-validation

Since there were two samples corresponding to two runs per each subject, another source of overly optimistic bias that we had to avoid was possible inclusion of the samples for the same subject in both training and test datasets - for example, if using the standard leave-one-out cross-validation approach. Instead, we used leave-one-subject-out cross-validation, where each of the 22 folds on the 44-sample dataset (11 schizophrenic and 11 control samples, 2 runs each) would set aside as a test set the two samples for a particular subject.

#### Controls

A potential artifact that affects the computation of functional connectivity networks is the movement of subjects in the scanner. While we implemented the standard procedure for movement correction, it is known that residual effects may still leave a trace in the functional images. For this we developed an approach that addresses the issue directly in the context of predictive modeling; specifically, we computed pairwise correlations between the movement parameters and each network feature, for all feature types listed in the paper (e.g., for degree features, we computed correlation between a motion parameter and degree of each voxel). To see whether those correlations were significant, we used the False Discovery Rate (FDR) test with significance level 0.05, on p-values corresponding to the correlations, and showed that there are no voxels that pass significance test (see Material S1). (FDR is a statistical method used in multiple hypothesis testing to correct for multiple comparison, discussed in more detail in the subsequent sections).

Moreover, we applied the same classification approach to data from alcoholic patients, acquired following the same protocol as normal and schizophrenic patients. Alcoholic patients are known to show a significant degree of movement inside the scanner. As reported in Material S1, the negative classification results are further indication that movement is not a factor.

## Results

### Model-driven ROI Analysis

First, we observed that correlation (blind to experimental paradigm) between regions and *within subjects* were very strong and significant (p-value of 0.05, corrected for the number of comparisons) when tested against 0 for all subjects (mean correlation >0.8 for every group). However, these inter-region correlations do not seem to differ significantly between the groups. The parcellation technique led to some smaller p-values, but also to a stricter correction for multiple comparison and no correlation was close to the corrected threshold. Concerning the psycho-physiological interaction, results were closer to significance, but did not survive multiple comparisons. In conclusion, we could not detect significant differences between the schizophrenic patient data and normal subjects in either the BOLD signal correlation or the interaction between the signal and the main experimental contrast (native language versus silence).

### Data-driven Analysis: Topological vs Activation Features

Empirical results are consistent with our hypothesis that schizophrenia disrupts the normal structure of functional networks in a way that is not derived from alterations in the activation; moreover, they demonstrate that topological properties are highly predictive, consistently outperforming predictions based on activations.

#### Voxel-level statistical analysis

In order to find out whether various features exhibit statistically significant differences across the two groups, we performed two-sample t-test for each feature 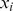 from the corresponding feature vector 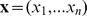 of a particular type (e.g., activations, degrees, etc.); herein, *n* is the number of voxels for voxel-level features, and 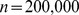 for the weight features (pairwise correlations). Clearly, when the number of statistical tests is very large (i.e., *n* here is exceeding 50,000), a correction for multiple comparisons is necessary, since low *p*-values indicating statistically significant differences given one test may just occur due to pure chance when many such tests are performed. A commonly used Bonferroni correction is overly conservative in brain imaging analysis since it assumes test independence, while there are obviously strong correlations across the voxel-level features. A more appropriate type of correction that is now frequently used in fMRI analysis is the False Discovery Rate (FDR) method, designed to control the *expected proportion* of incorrectly rejected null hypotheses, or “false discoveries”. In general, FDR is less conservative than the familywise error rate (FWER) methods (including the Bonferroni correction), since it does not guarantee there are *no false positives*, but rather that there are *only a few of them*. For example, FDR with threshold 0.05 guarantees no more than 5% of false positives. Herein, we include the results for both FDR and Bonferroni corrections (see columns 5 and 6 of the Table 1, respectively). However, our discussion is mainly based on FDR results, while Bonferroni results are mentioned purely for completeness sake, to demonstrate that some of the statistical differences we observed are so strong that they survived even an overly strict Bonferroni correction.

**Table 1:** Detailed t-test results for all activation and network-based features: each column shows the number of voxels that satisfy a given constraint, such as having p-value below the specified threshold or surviving the FDR or Bonferroni correction *with the significance level 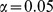
* (the number of voxels common with the full degree maps is shown in parenthesis for unnormalized linear activation maps).

map	*p*<0.01	*p*<0.001	*p*<0.0001	FDR	Bonferroni	N
norm. full degrees	2583	1046	448	1033	50	53750
norm. long-dist. deg.	2335	972	398	924	43	53737
norm. inter-hem. deg	1448	677	258	508	18	51373
activation 1 (3)	1799 (341)	317 (76)	52 (9)	7 (2)	0	53456
activation 2 (4)	805 (27)	112 (0)	15 (0)	0 (0)	0	53456
activation 5	1356 (306)	262 (69)	63 (10)	0 (0)	0	53456
activation 6	1481 (152)	303 (14)	55 (1)	2 (0)	1	53456
activation 7	1294 (130)	163 (13)	20 (1)	0 (0)	0	53456
activation 8	2369 (97)	467 (1)	53 (0)	0 (0)	0	53456
norm. activation 1 (3)	885	108	15	0	0	53456
norm. activation 2 (4)	688	95	13	0	0	53456
norm. activation 5	647	58	8	0	0	53456
norm. activation 6	1357	245	37	0	0	53456
norm. activation 7	1019	123	10	0	0	53456
norm. activation 8	1511	236	30	1	1	53456
corr subset(200 K)	23573	6437	1718	12240	37	199998
Strength	10917	2197	393	11294	6	53750
absolute strength	6721	1053	154	971	0	53750
positive strength	8938	1594	277	5724	2	53750
clustering coef.	3812	955	240	789	4	53750
local efficiency	4142	1076	286	1077	4	53750

The last column shows the total number of voxels *N* with non-zero values in the corresponding map (recall that Bonferroni correction filters out the voxels with 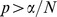). Note that for the activation maps, the results for both normalized and unnormalized maps are shown, since unnormalized ones performed better in hypothesis testing.

**Figure 4:**
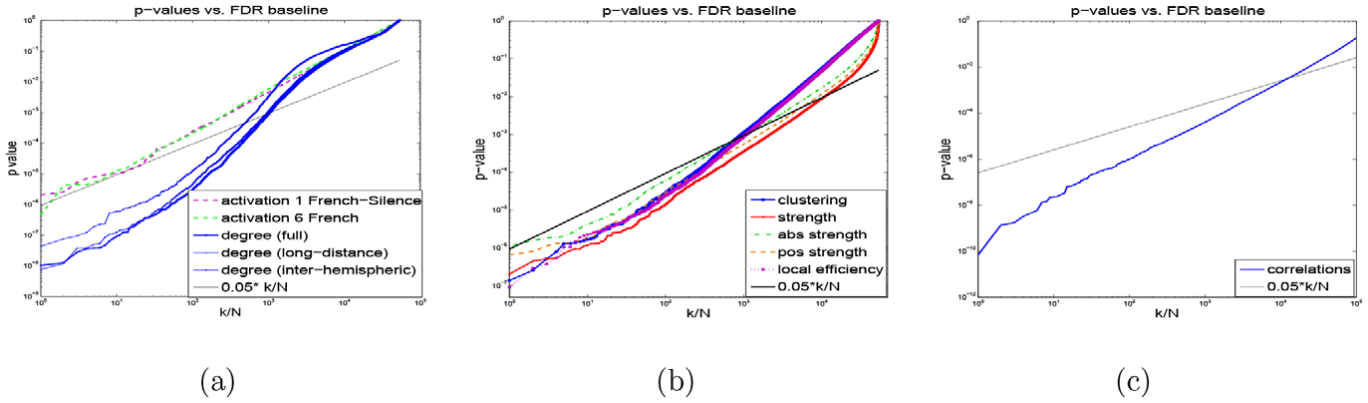
Two-sample t-test results for different features: p-values vs. FDR threshold. (a) Activations vs. normalized degrees; (b) clustering coefficients, strength, absolute strength, positive strength, and local efficiency of each voxel; (c) 200,000 randomly selected pairwise correlations. The null hypothesis for each feature assumes no difference between the schizophrenic vs normal groups. P-values of the features are sorted in ascending order and plotted vs FDR baseline; FDR test select voxels with 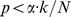, 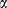 - false-positive rate, 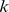 - the index of a p-value in the sorted sequence, *N* - the total number of voxels. Note that graph-based features yield a large number of highly-significant (very low) p-values, staying far below the FDR cut-off line, while only a few voxels survive FDR in case of (unnormalized) activation maps in panel (a): 7 and 2 voxels in activation maps 1 (contrast “FrenchNative – Silence”) and 6 (“FrenchNative”), respectively, while the rest of the activation maps do not survive the FDR correction at all.

Our main observation is that the *network features show much stronger statistical differences between the schizophrenic vs. non-schizophrenic groups than the activation features*. Figure 4 shows the results of two-sample t-test analysis for all voxel-level features, and the corresponding FDR threshold at 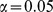 level. Panel (a) shows a direct comparison between the best activation features (dashed lines) and three (spatially normalized) degree maps: full, long-distance and inter-hemispheric. In all degree maps, on the order of 10^3^ voxels survive FDR correction (i.e., have their p-values below the black line corresponding to the FDR threshold), while only a handful (less than 10) of activation voxels do. The other measured graph features, including clustering and local efficiency, have less statistical power than degrees (i.e., have p-values closer to the FDR threshold), but yet outperform activation maps by almost two orders of magnitude, as shown in Panel (b). A full list showing the number of surviving voxels for each map is shown in Table 1. (Note that for the activation maps, the results for both normalized and unnormalized maps are shown, since unnormalized ones performed better in hypothesis testing. In classification study presented next, the situation was reversed, i.e. normalized activations predicted better than unnormalized; thus, we always included the best possible results achieved by activations. In case of degree maps, we always used only their normalized versions, which performed best in both hypothesis testing and classification scenarios.) Finally, randomly selected pairwise correlations, as shown in Panel (c), behave similarly to degrees, with an order of 10^4^ correlations surviving the FDR test, i.e. an order of magnitude more than for degrees. (Note, however, that the total number of correlation features (200,000) is also much larger than the number of degree features (about 50,000), i.e. voxels; therefore, the results for correlations are not directly comparable to those for degrees and other voxel features, and thus plotted in a separate panel.).

**Figure 5:**
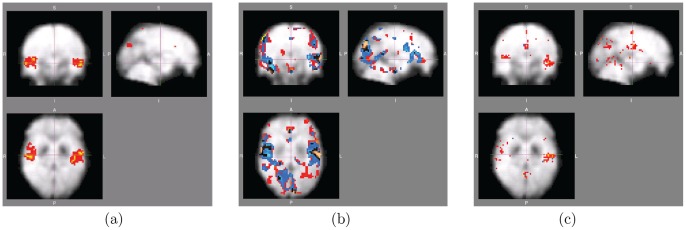
Two-sample t-test results for different features: voxels surviving FDR correction. (a) Normalized degree maps; (b) strength (red-yellow), absolute strength (blue-light blue) and positive strength (black-white); (c) clustering coefficient and local efficiency maps. Here the null hypothesis at each voxel assumes no difference between the schizophrenic vs normal groups. Colored areas denotes low p-values passing FDR correction at 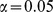 level (i.e., 5% false-positive rate). Note that the mean (normalized) degree at highlighted voxels was always (significantly) *higher* for normals than for schizophrenics. Coordinates of the center of the image: (a) and (c) X = 26,Y = 30,Z = 16, (b) X = 26,Y = 30,Z = 18.xl.

The spatial localization of the network maps is shown in Figure 5, representing the voxels surviving correction for (a) (normalized) degree maps, (b) strength (red-yellow), absolute strength (blue-light blue) and positive strength (black-white), (c) clustering coefficient and local efficiency maps. Normalized degrees (a) show the most spatially coherent organization, with contiguous bilateral clusters in auditory/temporal areas, prominently BA 22 and BA 21. Note also that the degree of the normal population is *higher* than the patient population. Strength-related features (b) have less bilateral symmetry and are also less spatially coherent, while clustering (c) is even more scattered.

**Figure 6:**
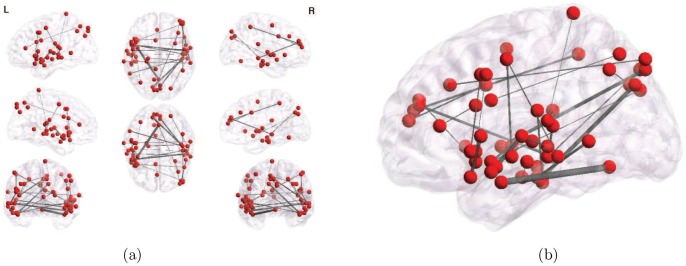
Thirty top-ranked (lowest-p-value) edges (all surviving Bonferroni correction) out of 200,000 pairwise correlation features, computed on the full dataset. (a) All views and (b) enlarged saggital view. Edge density is proportional to their absolute value.

**Figure 7:**
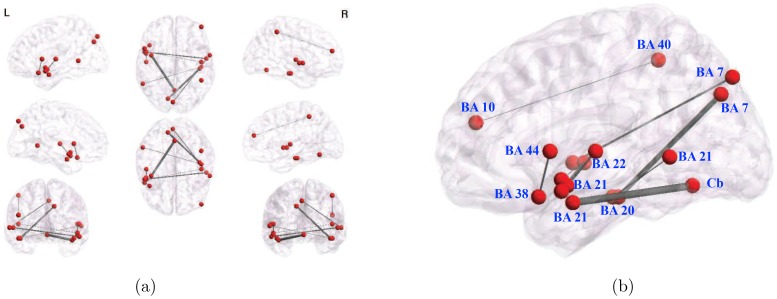
9 stable edges common to all subsets of 30 top-ranked (lowest-pvalue) edges that survived Bonferroni correction, over 22 different cross-validation folds (leave-subject-out data subsets). (a) All views and (b) enlarged saggital view. Edge density is proportional to their absolute value. The network includes several areas not picked up by the degree maps, i.e. other than BA 22 and BA 21, mainly the cerebellum (declive) and the occipital cortex (BA 19).

The network in Figure 6 visualizes the top 30 most significantly different edges selected out of 200,000 edge features, or pairwise correlations (the total number of such features surviving FDR correction was 12240, as shown in Table 1 and visualized in Figure 4b). Figure 7 shows a stable subset of 9 edges common to all top-30 ranked edges, over all cross-validation subsets, making it a highly robust representation. Note that unlike the degree maps, this network includes areas other than BA 22 and BA 21, prominently left precentral gyrus BA 44 (Broca’s area), right middle frontal gyrus BA 10, medial precuneus BA 7, and the declive of the cerebellum. A complete list of the nodes is presented in Table 2, while area-to-area functional connections determined by the 9 most stable links are shown in Table 3. Note that most links span both hemispheres, and that there are no local, intra-area links, even though we introduced no voxel clustering.

**Table 2:** Areas corresponding to the nodes on the 9 most stable links.

Hemis.	Broad Anatomy	Brodmann	x	y	z
R	Temporal Fusiform Gyrus	20	45	−24	−18
R	Temporal Fusiform Gyrus	20	48	−21	−18
L	Middle Temporal Gyrus	21	−42	0	−21
L	Middle Temporal Gyrus	21	−54	6	−15
L	Middle Temporal Gyrus	21	−51	2	−12
L	Middle Temporal Gyrus	21	−57	−51	3
L	Superior Temporal Gyrus	38	−45	18	−18
L	Superior Temporal Gyrus	38	−51	6	−9
R	Superior Temporal Gyrus	22	57	−6	0
R	Superior Temporal Gyrus	22	63	0	0
R	Superior Temporal Gyrus	22	48	−12	6
L	Superior Temporal Gyrus	22	−51	−12	6
L	Precentral Gyrus	44	−54	12	6
R	Middle Frontal Gyrus	10	48	51	21
L	Medial Precuneus	7	−12	−78	36
L	Medial Precuneus	7	−3	−84	45
R	Inferior Parietal Lobe	40	48	−45	54
-	Declive	Cb	0	−63	−12

**Table 3:** Area-to-area functional connections determined by the 9 more stable links.

left BA 21	↔	Cb
right BA 20	↔	left BA 7
right BA 20	↔	left BA 21
left BA 38	↔	left BA 44
left BA 21	↔	right BA 22
left BA 38	↔	right BA 22
right BA 22	↔	medial BA 7
right BA 10	↔	right BA 40

Our observations suggest that (a) the differences in the collective behavior cannot be explained by differences in the linear task-related response, and that (b) topology of voxel-interaction networks is more informative than task-related activations, suggesting an abnormal degree distribution for schizophrenic patients that appear to lack hubs in auditory cortex, i.e., have significantly lower (normalized) voxel degrees in that area than the normal group, possibly due to a more even spread of degrees in schizophrenic vs. normal networks. Note that, as discussed earlier, ROI- and parcellation-level network topologies do not seem to retain information present in voxel-level networks, apparently due to averaging the signal over ROIs or parcels.

**Figure 8:**
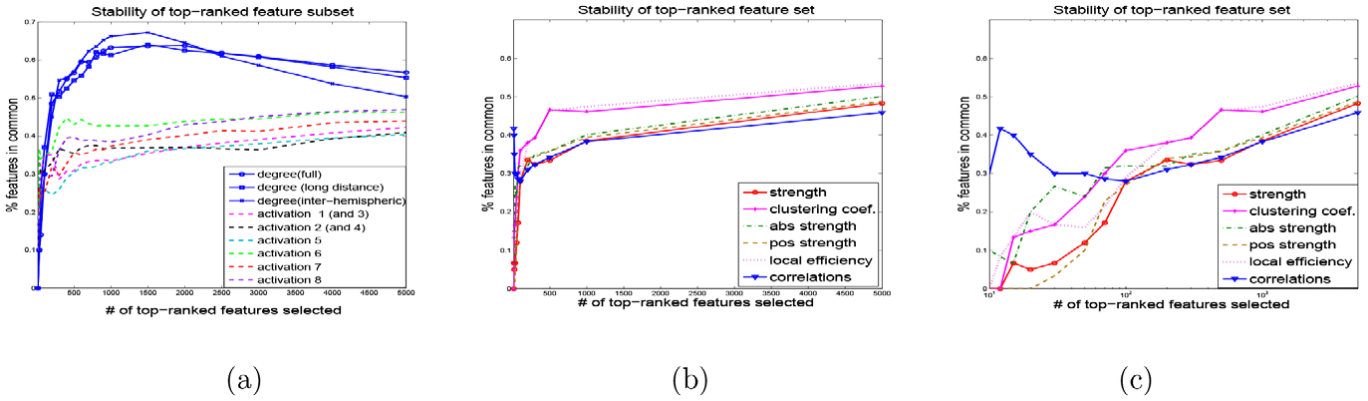
Stability of feature subset selection over cross-validation (CV) folds. Stability is measured as the percent of voxels in common among the subsets of *k* top variables selected at all CV folds: (a) activations and degrees; (b,c) edge weights (correlations), clustering coefficients, strength, absolute strength, positive strength, and local efficiency: (b) linear scale on x-axis, (c) log-scale on x-axis (focusing on small number of features selected.

We also evaluate the *stability* of all features with respect to selecting a subset of top ranked voxels over different subsets of data. For each value of 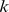, stability of the top-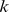-ranked feature subset is defined as a fraction of features in common over all cross-validation data subsets (recall that there are 22 of them). Namely, given a fixed value of 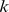, for each data subset, we rank the features by their 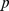-values computed on that particular subset, choose the top *k* of them, and then compute the intersection over all 22 of those top- *k* feature subsets. The number of features common to all subsets (i.e., the size of their intersection), divided by *k*, gives us a measure of feature stability. Interestingly, network-based features, such as degrees (full, long-distance or inter-hemispheric) demonstrate much *higher stability* than activation features, as well as other network-based features. Figure 8a shows that degree maps have up to almost 70% top-ranked voxels in common over different training data sets when using the leave-one-subject out cross-validation, while activation maps have below 50% voxels in common between different selected subsets. This property of degree vs activation features is particularly important for interpretability of predictive modeling. Stability of the other network-based features is shown in Figures 8b and 8c, where the Figure 8c shows the same results as Figure 8b, but using logarithmic scale instead of linear, in order to focus on the regimes when only a small number of features is selected. While the overall stability of the remaining network features does not reach the high values of the degree features, it is still interesting to note that the pairwise correlations appear to be the most stable of the remaining network features when the number of selected features is relatively small, e.g. below 100.

#### Inter-hemispheric degree distributions

**Figure 9:**
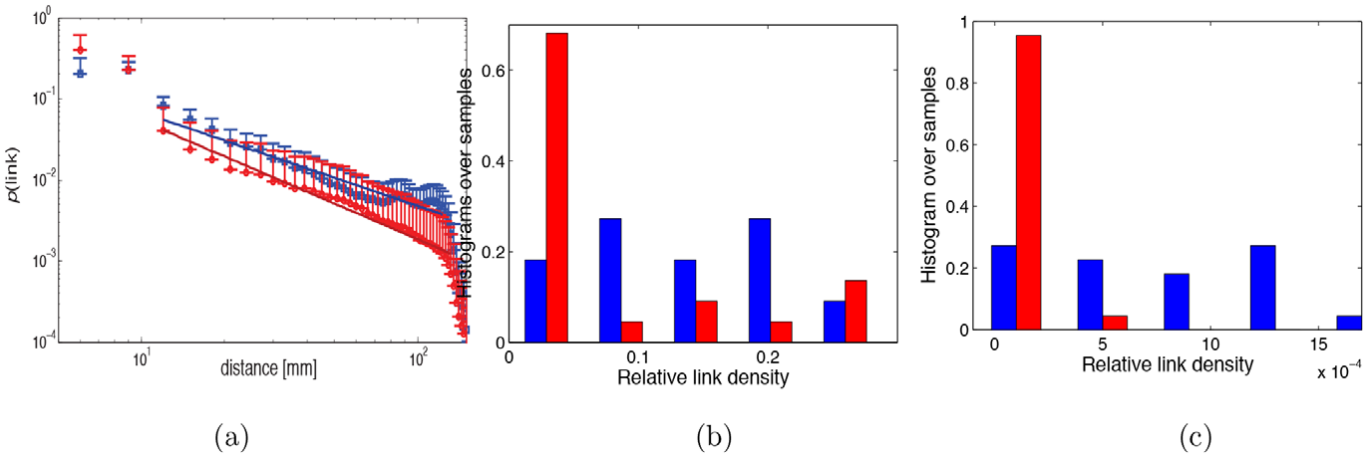
Functional connectivity disruption in schizophrenic subjects vs controls. (a) Probability of finding a network link as a function of the Euclidean distance between the nodes (in millimeters): schizophrenics (red) show reduced connectivity than controls (blue) for distances in the middle range (10 to 150 mm). (b) Disruption of *global* inter-hemispheric connectivity. For each subject, we compute the fraction of links spanning both hemispheres over the total number of links, and plot a normalized histogram over all subjects in each group (normal - blue, schizophrenic - red). (c) Disruption of *task-dependent* inter-hemispheric connectivity between specific ROIs (Brodmann Area 22 selected bilaterally). The ROIs were defined by a 9 mm radius ball centered at [x = −42, y = −24, z = 3] and [x = 42, y = −24, z = 3]. For each subject, we compute the fraction of links connecting the bilateral ROIs over all links, and show a histogram of this connectivity measure over all subjects in each group. The histograms are similarly normalized.

As suggested by the predominance of inter-hemispheric edges in the set of most significantly different pairwise correlations (Table 3), a closer look at the degree distributions reveals that a large percentage of the differential connectivity appears to be due to long-distance, inter-hemispheric links. Figure 9a compares the probability of finding a link in the networks as a function of the Euclidean distance between the nodes (in millimeters), for schizophrenic (red) versus control (blue) subjects. The bars correspond to one standard deviation, drawn on the top only, to avoid clutter in the figure, and the lines correspond to power-law fits for the intermediate distances (i.e. between 10 and 150 mm). The fit is 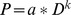, with 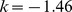 for schizophrenics, and 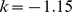 for controls. We see that for this distance range, schizophrenics have reduced connectivity, i.e. lower link probabilities than controls. Figure 9b compares the fraction of inter-hemispheric connections over all connections, for schizophrenic (red) versus normal (blue) groups. For each subject, a unique value was computed dividing the number of links spanning both hemispheres by the total number of links. The figure represents the normalized histogram of this inter-hemispheric link density for each group. The schizophrenic group shows a significant bias towards low relative inter-hemispheric connectivity. A t-test analysis of the distributions indicates that differences are statistically significant (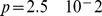). Moreover, it is evident that a major contributor to the high degree difference discussed before is the presence of a large number of inter-hemispheric connections in the normal group, which is absent in schizophrenic group. Furthermore, we selected a bilateral region of interest (ROI) corresponding to left and right Brodmann Area 22 (roughly, the clusters in Figure 4a), *such that the linear activation for these ROI’s was not significantly different between the groups*, even in the uncorrected case. For each subject, the connection strength between the left and right ROIs was computed as the fraction of ROI-to-ROI links over all links. Figure 9c shows the normalized histogram over subjects for this connectivity measure. Clearly, the normal group displays higher ROI-to-ROI connectivity, which is significantly disrupted in the schizophrenic group (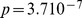). This provides a strong indication that the group differences in connectivity cannot be explained by differences in local activation.

#### Global features

For each global feature (full list in Material S1) we computed its mean for each group and p-value produced by the t-test, as well as the classification accuracies using our classifiers. While more details are presented in Material S1, we outline here the main observations: while mean activation (we used map 8, the best performer for SVM on the full set of voxels - see Table 4b) had a relatively low p-value of 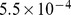, as compared to less significant 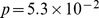 for *mean-degree*, the predictive power of the latter, alone or in combination with some other features, was the best among global features reaching 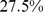 error in schizophrenic vs normal classification (Table 4a), while mean activation yielded more than 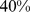 error with all classifiers In general, low p-values not necessarily imply low generalization error, as the results with other global features show. This is not particularly surprising, especially when the data violate Gaussian assumption of the t-test as it is in our case.

**Table 4:** Classification errors using (a) global features and (b) activation and degree maps (using SVM on the complete set of voxels (i.e., without voxel subset selection).

(a)			
Feature	GNB	SVM	MRF(0.01)
degree (D)	27.5%	27.5%	27.5%
clustering coeff. (C)	30.0%	42.5%	45.0%
geodesic dist. (G)	67.5%	45.0%	45.0%
mean activation (*A*)	40.0%	45%	72.5%
D+A	27.5%	27.5%	32.5%
C+A	27.5%	45.0%	55.0%
G+A	45.0%	45.0%	72.5%
G +D +C	37.5%	27.5%	27.5%
G+D+C+A	30.0%	27.5%	32.5%
**(b)**			
**Feature**	**Err**	**FP**	**FN**
correlations (53750)	**14%**	**14%**	**14%**
degree (full)	**16%**	**27%**	**5%**
degree (long-distance)	**21%**	**32%**	**9%**
degree (inter-hemis)	32%	46%	18%
clustering	23%	32%	14%
local efficiency	23%	32%	14%
strength	23%	23%	23%
abs strength	34%	41%	27%
pos strength	25%	32%	18%
activation 1 (and 3)	54%	29%	82%
activation 2 (and 4)	50%	55%	45%
activation 5	43%	18%	68%
activation 6	36%	27%	46%
activation 7	32%	18%	46%
activation 8	30%	23%	37%

For each feature, we show the average error, as well as the fraction of false positives (FP) and false negatives (FN).

#### Classification using activations vs. network features

While mean-degree indicates the presence of discriminative information in voxel degrees, its generalization ability, though the best among global features and their combinations, is relatively poor. However, voxel-level network features turned out to be very informative about schizophrenia, often outperforming activation features by far. Table 4b shows the results of classification by SVM using all voxel-level network features of each type. Herein, all voxels and their corresponding features were used, without any subset selection; for correlation features, defined on pairs of voxels, we just used same number of features as in all other cases, i.e. the top 53750 correlations out of 200000, since 53730 is the number of voxels used in the other features. Note that the top-performing network features are correlations (14% error) and (full) degree maps (16% error), greatly outperforming all activation maps that yield above 30% error for even the best-performing activation map 8.

**Figure 10:**
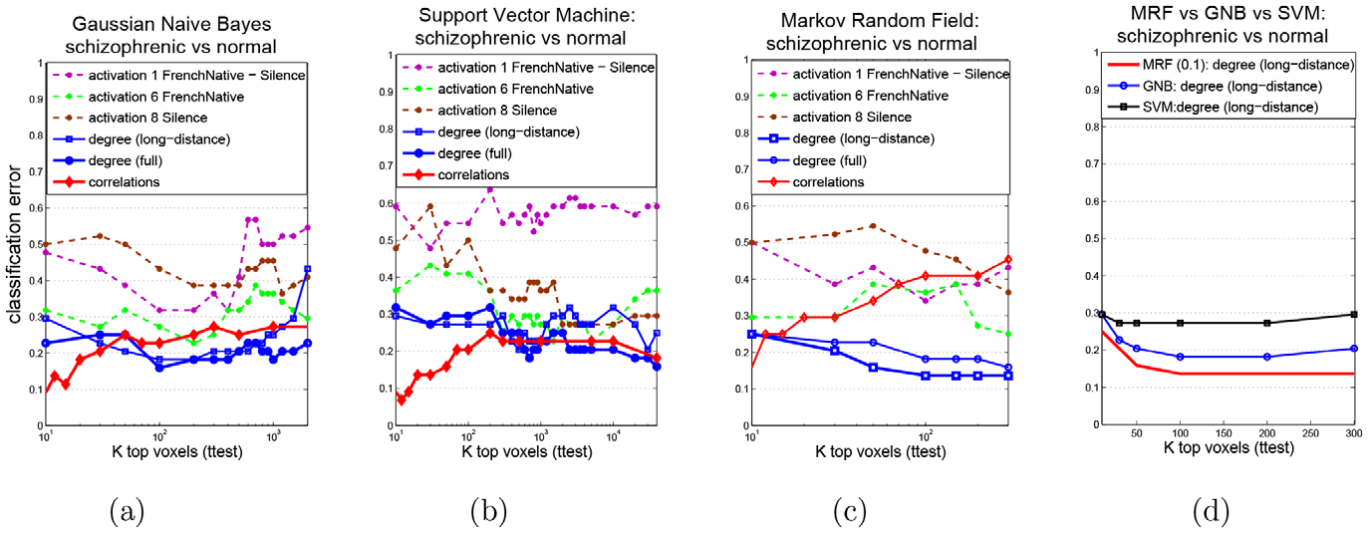
Classification results: degree vs. activation features. Three classifiers, Gaussian Naive Bayes (GNB) in panel (a), SVM in panel (b) and sparse MRF in panel (c) are compared on two types of features, degrees and activation contrasts; (d) all three classifiers compared on long-distance degree maps (best-performing for MRF).

Next, in Figure 10, we compare the predictive power of different features using all three classifiers: Support Vector Machines (SVM), Gaussian Naive Bayes (GNB) and sparse Gaussian Markov Random Field (MRF), on the subsets of *k* top-ranked voxels, for a variety of *k* values. For sparse MRF, we experimented with a variety of 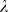 values, ranging from 0.0001 to 10, and present the best results; while cross-validation could possibly identify even better-performing values of 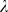, it was omitted here due to its high computational cost (also, using the fixed values listed above we already achieved quite high predictive accuracy as described later). We used the best-performing activation map 8 from the Table above, as well as maps 1 and 6 (that survived FDR); map 6 was also outperforming other activation maps in low-voxel regime. Also, to avoid clutter, we only plot the results for the three best-performing network features: full and long-distance degree maps, and pairwise correlations. Classification results for the rest of network features can be found in Appendix. We can see that:

- *Network features outperform activation maps*, for all classifiers we used, and for practically any value of *k*, the number of features selected. The differences are particularly noticeable when the number of selected voxels is relatively low.

The most significant differences are observed for SVM in low-voxel (approx. 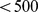) regime: using just a dozen of most-predictive pairwise correlations achieves a remarkable 7% error while the activation maps yield 30% and larger errors. Also, both pairwise correlations and degrees noticeably outperform activations on the full set of features (far right of the x-axis). Moreover, degree features demonstrate excellent performance with MRF classifiers: they achieve quite low error of 14% with only 100 most significant voxels, while even the best activation map 6 requires more than 200–300 to get just below 30% error; the other activation maps perform much worse, often above 30–40% error, or even just at the chance level.

- *Full and long-distance degree maps perform quite similarly*, with long-distance map achieving the best result (14% error) using MRFs.
- Among the activation maps only, while the map 8 (“Silence”) outperforms others on the full set of voxels using SVM, its behavior in low-voxel regime is quite poor (always above 30–35% error); instead, map 6 (“FrenchNative”) achieves best performance among activation maps in this regime. We also observed that performing normalization really helped activation maps, since otherwise their performance could get much worse, especially with MRFs - we provide those results in Material S1.
- *MRF classifiers significantly outperform SVM and GNB with degree features*, possibly due to their ability to capture inter-voxel relationships that are highly discriminative between the two classes (see Figure 10d). However, with the correlation features the situation is reversed, and the overall best results (7% error) is achieved using SVM with just a dozen of top-ranked correlations.

## Discussion

Attributing schizophrenia to abnormal interactions among different brain areas, rather than to local failures, has a long history in schizophrenia research, and is sometimes referred to as the “disconnection” hypothesis [22]. According to [23], this hypothesis was first proposed in 1906 by Wernicke [24], who postulated that anatomical disruption of association fiber tracts is at the roots of psychosis; in fact, the term “schizophrenia” was introduced by Bleuler [25] in 1911, and was meant to describe the separation (“splitting”) of different mental domains.

Recent advances in neuroimaging provided researchers with tools for studying not just anatomical, but also functional connectivity and its disruption in schizophrenia. The “disconnection syndrome” article by [22] was among the first ones to point out abnormalities in functional connectivity using PET imaging data (see also [26]). (More recently, the “dysconnection” term was suggested [23] in order to better capture the fact that schizophrenia is associated with a broader range of network dysfunctions besides just missing connections.) The paper studied functional connectivity captured by temporal correlations among different brain areas during a linguistic task, using principal component analysis (PCA) decomposition of the functional connectivity (covariance) matrix. Analysis of spatial components (“eigenimages”) revealed that “profound negative prefronto-superior temporal functional interactions associated with intrinsic word generation” was strongly present in healthy subjects, but practically absent in schizophrenic patients; vice versa, positive prefronto-left temporal correlations were present in schizophrenic group but in the normal group, suggesting a reversal of prefronto-temporal integrations, attributed to “failure of prefrontal cortex to suppress activity in the temporal lobes (or vice versa)”.

More recently, several studies demonstrated altered patterns in default-mode networks of schizophrenia, e.g. altered temporal frequency and spatial location of the default mode networks [5], and other patterns of aberrant connectivity [27], [28]. Also, multiple recent studies [7], [29] focused on graph-theoretic analysis of *functional connectivity networks* [8] in schizophrenia, demonstrating, for example, that in schizophrenia patients “the small-world topological properties are significantly altered in many brain regions in the prefrontal, parietal and temporal lobes” [7]. There is also continuing work exploring abnormalities in anatomical networks in schizophrenia [6], [30], [31].

In general, the importance of modeling brain connectivity and interactions became widely recognized in the recent neuroimaging literature beyond schizophrenia research ([32]–[34] give just a few examples). However, practical applications of such approaches such as dynamic causal modeling [32], dynamic Bays nets [33], or structural equations [34] are often limited to interactions among a relatively small number of known brain regions believed to be relevant to the task or phenomenon of interest. As discussed below, such approach can be sometimes disadvantageous, while a more data-driven, *voxel-level functional networks* analysis can achieve better results.

In this paper, we proposed an approach to constructing predictive features based on functional network topology, and applied it to predictive modeling of schizophrenia. We demonstrated that (1) specific *topological properties of functional networks yield highly accurate classifiers of schizophrenia* and (2) *functional network differences cannot be attributed to alteration of local activation patterns*, a hypothesis that was not ruled out by the results of [6], [7] and similar work. In other words, our observations strongly support the hypothesis that schizophrenia is indeed a *network disease*, associated with the disruption of global, emergent brain properties.

Specifically, we demonstrated that topological properties of (voxel-level) functional brain networks are highly informative about the disease, unlike localized, task-related voxel activations, that were greatly outperformed by network-based features in both hypothesis testing and predictive settings. We also showed that it is highly important to use functional networks at the proper level: in our study, discriminative information present in voxel-level networks was apparently lost (perhaps due to averaging over large groups of voxels) at both regions-of-interest (ROI) and functional parcellation levels; the latter did not reveal any statistically significant differences between the schizophrenic and control groups. Unlike most traditional studies of schizophrenia networks based solely on hypothesis testing approach (e.g., [6], [7], [31]), we also employed *predictive modeling* techniques in order to evaluate how well the models built using network vs. local features would *generalize* to previously unseen subjects. Using generalization power, besides statistical significance, provides a complimentary (and often a more accurate) measure of disease-related information contained in a particular type of features, such as network properties or local activations. Moreover, predictive models have potential applications in clinical setting, e.g. for early diagnosis of schizophrenia based on abnormal patterns in imaging data. (Note, however, that multiple studies on a variety of subjects and experimental conditions may be necessary to come up with a robust predictive model).

In summary, our observations suggest that *voxel-level functional networks may contain significant amounts of information discriminative about schizophrenia, which may not be otherwise available in voxel activations or ROI-level networks*. Note, however, that the schizophrenic population studied here has been selected for their prominent, persistent, and pharmaco-resistant auditory hallucinations [20], which might have increased its clinical homogeneity and reduced its value as representative of the full spectrum of the disease. The experimental protocol may also restrict the applicability of our approach to generic cases. The areas more evidently involved in the discriminative networks, BA 22 and BA 21, are involved in language processing and are known to alter their activity in schizophrenics [35], and to display genetic and anatomical anomalies [36]. The direct analysis of pairwise correlations (as opposed to the voxel-centric degree maps) identifies anomalies in functional connectivity with Broca’s area, the cerebellum and, interestingly, the frontal lobe (BA 10), in loose agreement with previous findings regarding disrupted fronto-temporal connectivity associated with auditory hallucinations [37]. However, the analysis of correlations as a function of (Euclidean) distance provides a more nuanced perspective, as it shows weaker long-distance and stronger short-distance correlations for the patient population. This suggests a global re-organization of functional connections, and is further evidence of the emergent nature of the disruptions introduced by the disease. In the context of this finding, the identification of specifically affected areas, or area-to-area links, may be less relevant for the purpose of understanding functional alterations.

Note that the hypothesis of an emergent signature for schizophrenia does not necessarily reject the possibility of localized activation differences with respect to the normal population, for specific tasks or conditions. The finding that long-range functional connections are differentially affected, as demonstrated by the paucity of inter-hemispheric links and the weakness of long-distance correlations, may still be interpreted in terms of localized changes. Our findings may follow from subtle, undetectable changes (by fMRI at least) in the local activation of a handful of areas, that get amplified by the effect of the large number of links that are pooled when network features are computed, and bear no relationship to disruptions in the effective connectivity of the network (determined, for instance, by the lack or excess of specific neuro-transmitters). The fact is, however, that there is no such thing as a completely “local” activation in the brain, since the driving input to most areas of the central nervous system is provided by the activity of other areas. In this sense, the hypothesis can be reformulated to imply that the disease is concomitant with a much stronger disruption of emergent than of local features.

While our conclusions may not necessarily apply to the schizophrenic population in general, we believe that our approach transcends the specific details of the particular population and experimental protocol we studied, and can guide future investigations of schizophrenia and other complex psychiatric diseases that can be better understood as network dysfunctions. Directions for further research include exploration of network abnormalities in other schizophrenia studies that involve different groups of patients and different tasks, as well as better characterization of connections involved in the predictive discrimination.

## Supporting Information

Figure S1 **Demonstration of connectivity-based vs. locally-based changes in correlation for coupled oscillators.** The upper panels show the effect of changing the coupling strength of the oscillators, leading to drastic changes in correlation that do not affect the rates. The lower panels show the effect of changing the intrinsic rate of one oscillator while keeping the connection strength fixed. The correlation also changes drastically, but the change is associated with a change in the rate.
(TIF)

Figure S2 **Demonstration of connectivity-based vs. locally-based changes in correlation for Ising spins.** By changing the local field *h*, it is possible to affect the correlation while keeping constant the coupling parameter *J* (black arrow).
(TIF)

Figure S3 **Classification results comparing GNB, SVM and sparse MRF classifiers on** *unnormalized* **(raw) activation maps vs degree maps.**
(TIF)

Figure S4 **Classification results comparing (a) GNB, (b) SVM and (c) sparse MRF on correlations, clustering coefficient and strength features.**
(TIF)

Figure S5
**FDR-corrected 2-sample t-test results showing p-values associated with correlations between different features and the movement parameter.** The following features are presented: (a) pairwise voxel correlations (edge weights) (b) voxel-wise network features; (c) activations. The null hypothesis assumes no (significant) correlation between the feature and the movement parameter. P-values for each feature-movement correlation are sorted in ascending order and plotted vs FDR baseline; FDR test select voxels with 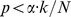, 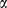 - false-positive rate, *k* - the index of a p-value in the sorted sequence, *N* - the total number of tests. Note that practically no p-value survives the FDR correction, suggesting that correlations between the features and the movement parameter are not statistically significant.(TIF)

Figure S6 **Results for schizophrenic vs (normal+alchoholic) classification.**
(TIF)

Table S1 Global features.
(TIF)

Table S2 Classification errors using global features schizophrenics vs. normal+alcoholics, baseline error about 31%.
(TIF)

Materials S1 **Supplemental materials.**
(PDF)
