# Expression of Concern: Schizophrenia as a Network Disease: Disruption of Emergent Brain Function in Patients with Auditory Hallucinations

**DOI:** 10.1371/journal.pone.0345144

**Published:** 2026-03-18

**Authors:** The PLOS One Editors

After this article [1] was published, the *PLOS One* Editors identified concerns about the peer review of [1]. We regret that the issues were not addressed prior to the publication of [1].

In light of the concerns which were not resolved in our discussions with the authors, the *PLOS One* Editors issue this Expression of Concern. Readers are advised to interpret the article with caution.
