# Supplementary figures and images for: Schizophrenia as a Network Disease: Disruption of Emergent Brain Function in Patients with Auditory Hallucinations
Source: PLoS One. 2013 Jan 21;8(1):e50625. doi: 10.1371/journal.pone.0050625 (PMC3549920; doi:10.1371/journal.pone.0050625)

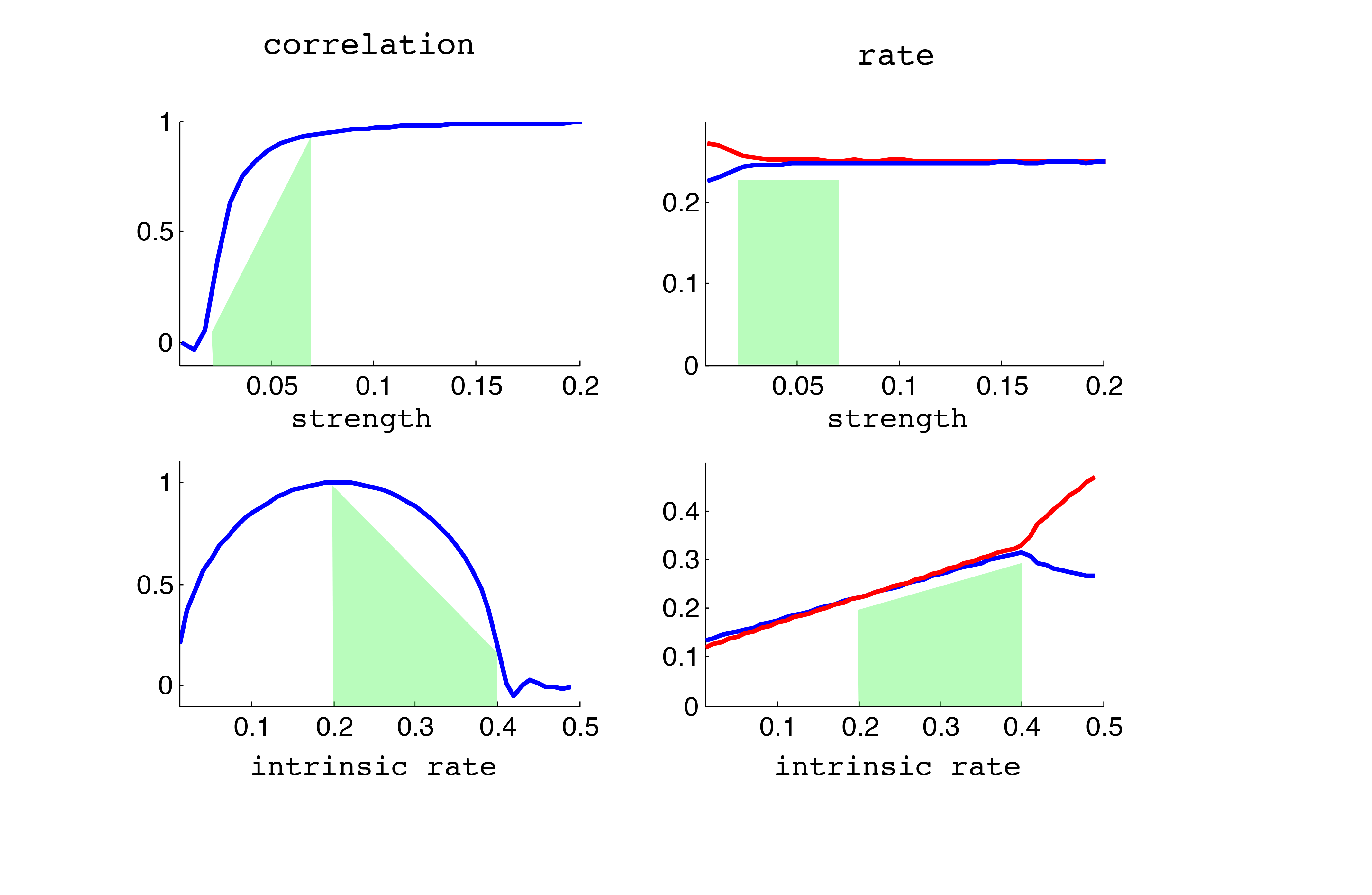

Supplement: Figure S1 — Demonstration of connectivity-based vs. locally-based changes in correlation for coupled oscillators. The upper panels show the effect of changing the coupling strength of the oscillators, leading to drastic changes in correlation that do not affect the rates. The lower panels show the effect of changing the intrinsic rate of one oscillator while keeping the connection strength fixed. The correlation also changes drastically, but the change is associated with a change in the rate. (TIF) [file pone.0050625.s001.tif]

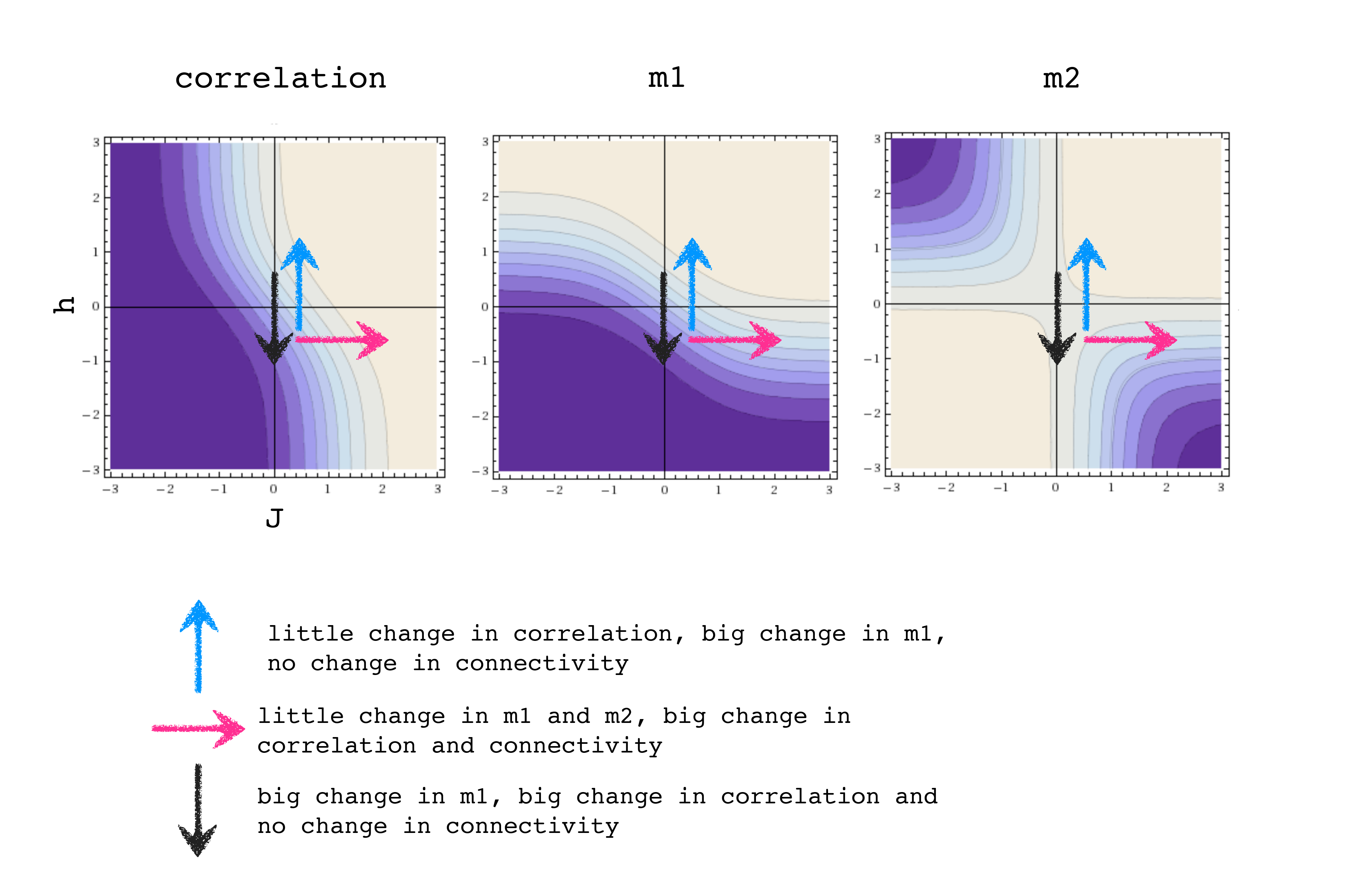

Supplement: Figure S2 — Demonstration of connectivity-based vs. locally-based changes in correlation for Ising spins. By changing the local field h, it is possible to affect the correlation while keeping constant the coupling parameter J (black arrow). (TIF) [file pone.0050625.s002.tif]

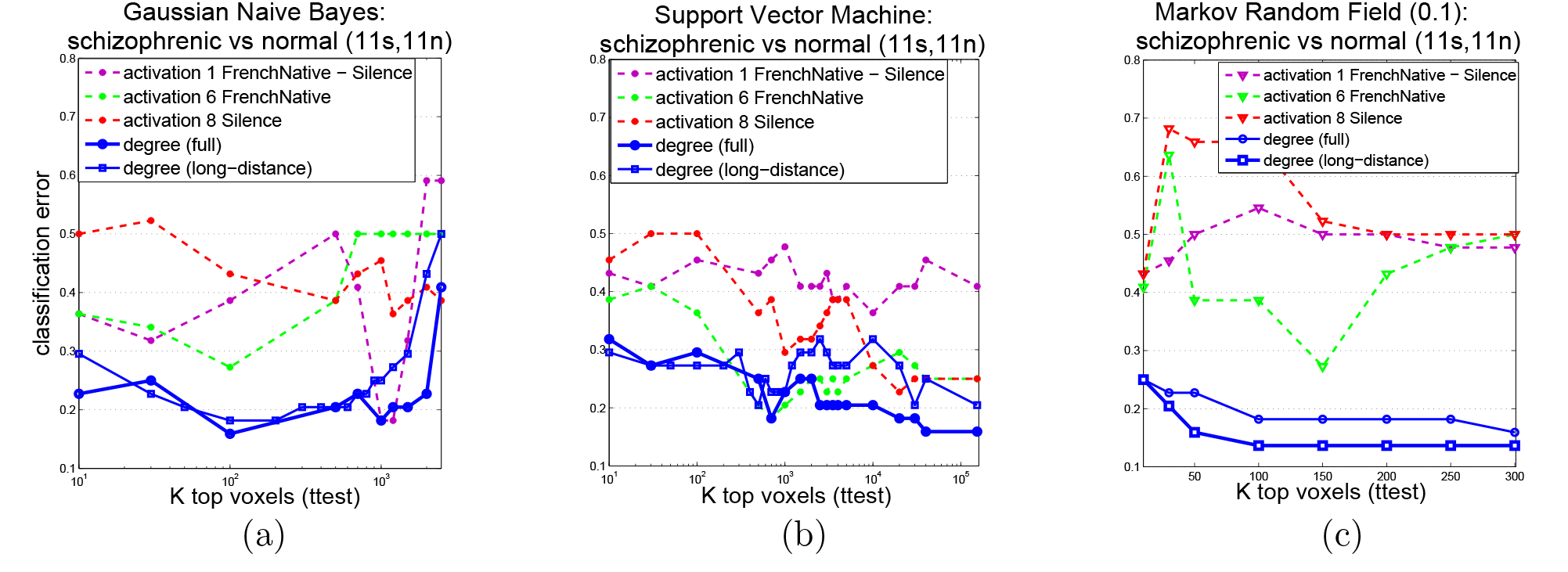

Supplement: Figure S3 — Classification results comparing GNB, SVM and sparse MRF classifiers on unnormalized (raw) activation maps vs degree maps. (TIF) [file pone.0050625.s003.tif]

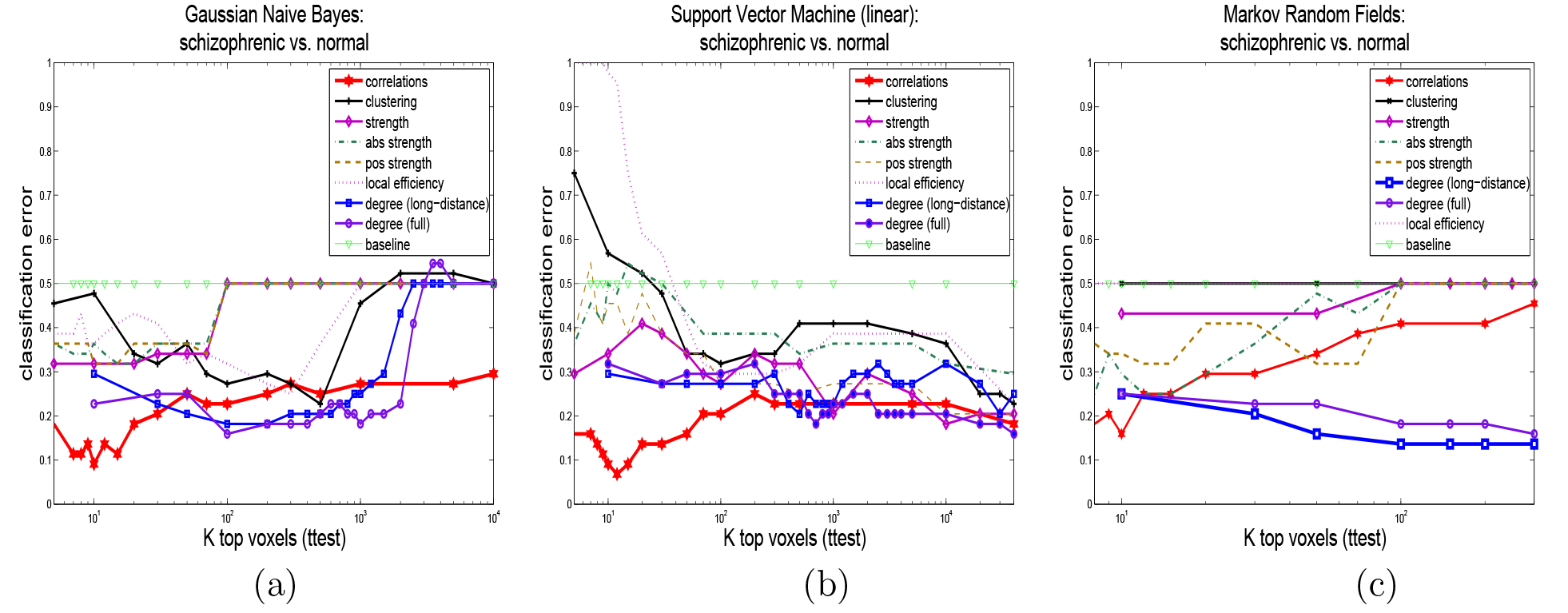

Supplement: Figure S4 — Classification results comparing (a) GNB, (b) SVM and (c) sparse MRF on correlations, clustering coefficient and strength features. (TIF) [file pone.0050625.s004.tif]

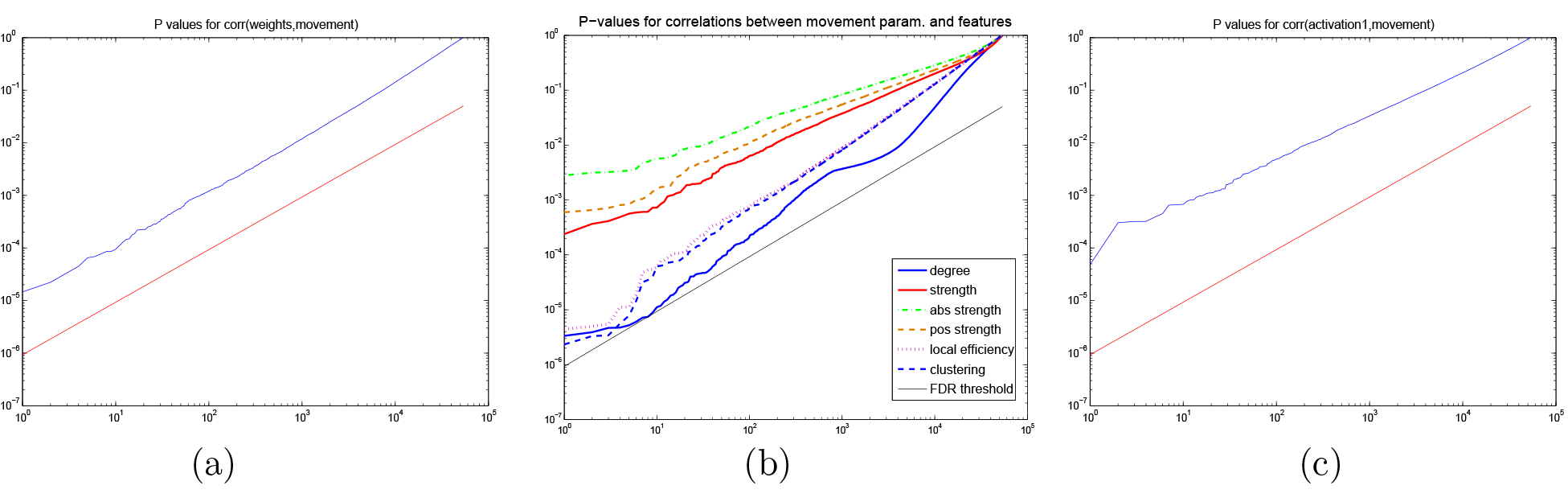

Supplement: Figure S5 — FDR-corrected 2-sample t-test results showing p-values associated with correlations between different features and the movement parameter. The following features are presented: (a) pairwise voxel correlations (edge weights) (b) voxel-wise network features; (c) activations. The null hypothesis assumes no (significant) correlation between the feature and the movement parameter. P-values for each feature-movement correlation are sorted in ascending order and plotted vs FDR baseline; FDR test select voxels with , - false-positive rate, k - the index of a p-value in the sorted sequence, N - the total number of tests. Note that practically no p-value survives the FDR correction, suggesting that correlations between the features and the movement parameter are not statistically significant. (TIF) [file pone.0050625.s005.tif]

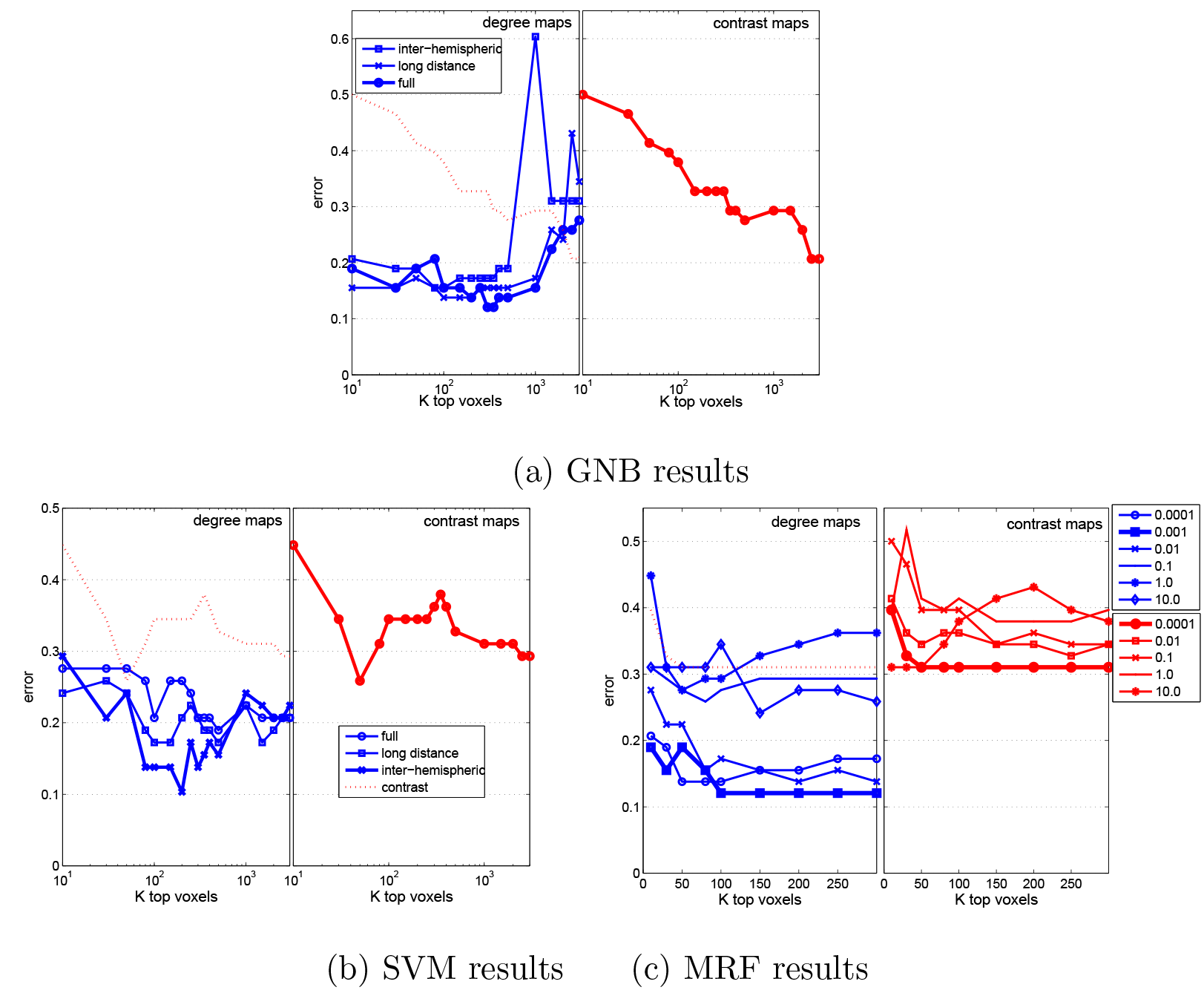

Supplement: Figure S6 — Results for schizophrenic vs (normal+alchoholic) classification. (TIF) [file pone.0050625.s006.tif]

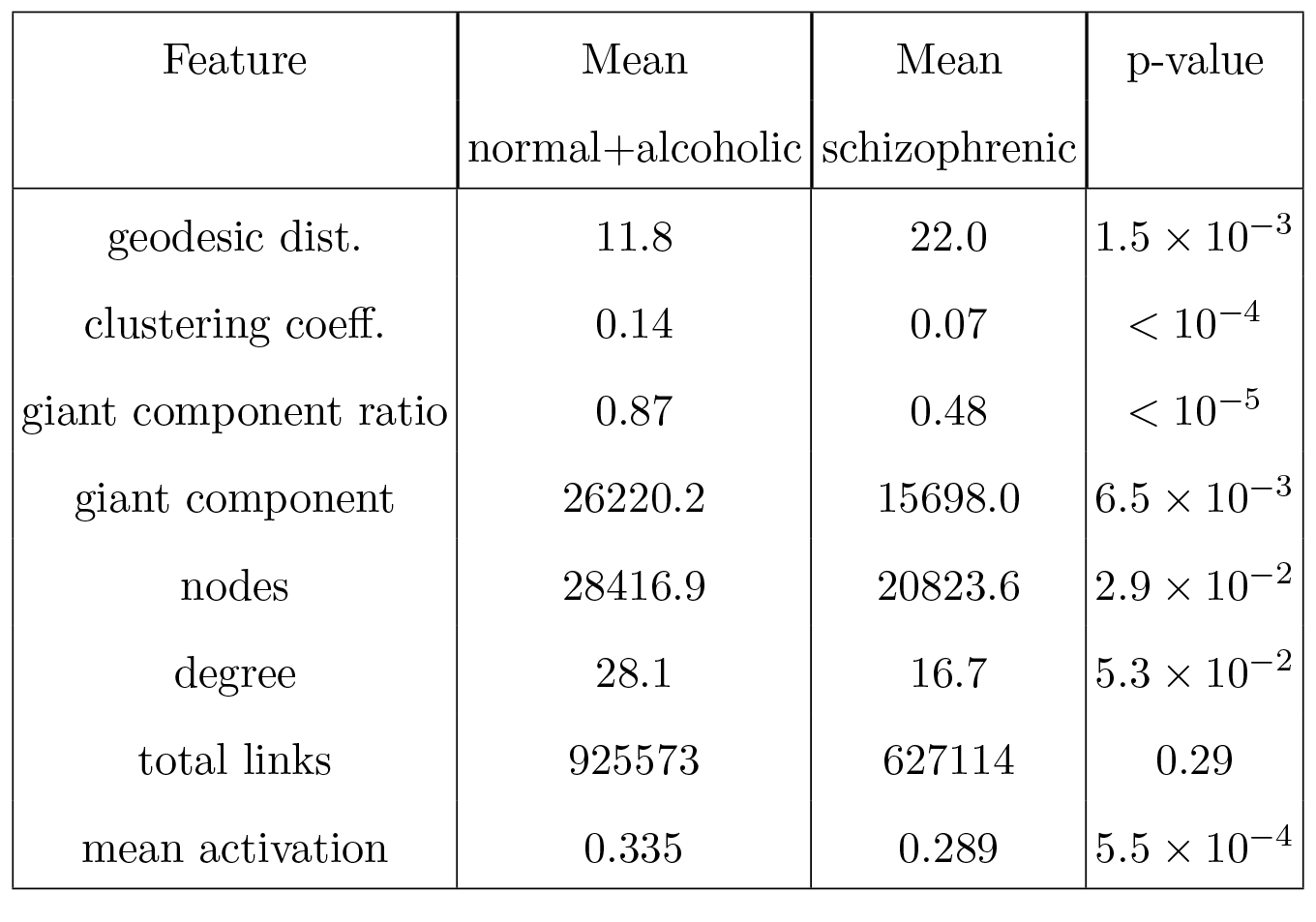

Supplement: Table S1 — Global features. (TIF) [file pone.0050625.s007.tif]

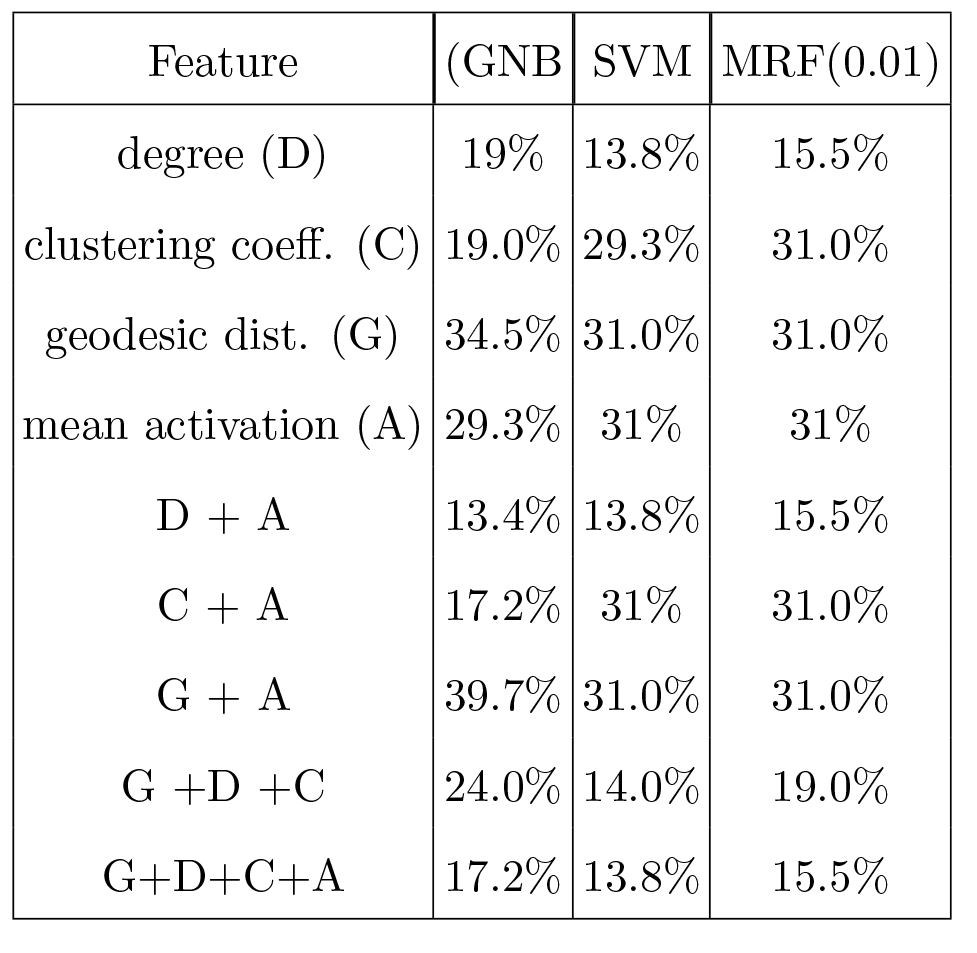

Supplement: Table S2 — Classification errors using global features schizophrenics vs. normal+alcoholics, baseline error about 31%. (TIF) [file pone.0050625.s008.tif]
