# Supplementary material for: Schizophrenia as a Network Disease: Disruption of Emergent Brain Function in Patients with Auditory Hallucinations
Source: PLoS One. 2013 Jan 21;8(1):e50625. doi: 10.1371/journal.pone.0050625 (PMC3549920; doi:10.1371/journal.pone.0050625)
Supplement: Materials S1 — Supplemental materials. (PDF) [file pone.0050625.s009.pdf]

Supplemental Material S1

Coupling Models

We argue that a network effect that is correlated with a change in the local activation is qualitatively different from one that does not correlated with significant changes in the local activation. In the first case, the distributed effect may be simply explained by the change in the local activation. Given the interconnected nature of the brain, it is almost unavoidable that a strictly local change will propagate as a change in the activation of other areas of the brain.

On the other hand, a change in the true connectivity of the network (adding or removing links, changing the connection weights) may not necessarily affect the local activity in terms of rates (i.e. first-order statistics) but should change the structure of cross-correlations (multi-point statistics).

We exemplify this first with a classical model of couple oscillators, the Kuramoto model. The oscillators are described by a phase, a natural or intrinsic rate of phase change (frequency), and a coupling. The coupling is implemented through a sine function on the phase difference between the local and input units, and can have varying strength. The pioneering work of Wilson and Cowan demonstrated that under very general conditions, neural tissue is expected to behave as a field of oscillators isomorphic to Kuramoto's model. A typical behavior is that, assuming that the units also have a certain level of noise, for a given difference between the intrinsic rate of the units, a small coupling strength will fail to synchronize the units.

For the purpose of illustrating our point, we simulated two units, with the following dynamics for $\theta(t)$:

$$\begin{aligned} \dot{\theta}_1 &= \omega_1 + K\sin(\theta_2 - \theta_1) + \eta_1(t) \\ \dot{\theta}_2 &= \omega_2 + K\sin(\theta_1 - \theta_2) + \eta_2(t) \end{aligned} \tag{1}$$

where ω is the intrinsic rate, K the coupling (assumed symmetric) and $\eta(t)$ gaussian noise. The results are shown in Figure S1. The two upper panels show the correlation between the oscillators, defined as $c = \langle\cos\theta_1\cos\theta_2\rangle/\sqrt{\sigma_1\sigma_2}$, $\sigma_i = \langle\cos^2\theta_i\rangle$ (left panel) and the individual rates, defined as $r_i = \langle\dot{\theta}_i\rangle$ (right panel), as a function of the connection strength K. The intrinsic rates for the oscillators are fixed at 0.2 and 0.25. As it can be readily seen, in the range of strength highlighted by the green shading, a change in the connectivity produces a drastic change in correlation, without a significant change in the effective rate of the oscillators. Conversely, the two lower panels show the result of changing the rate of one oscillator without changing the coupling strength. We fixed the coupling at 0.1 and the rate of one oscillator at 0.2, while changing the rate of the other from 0.01 to 0.5. The simulation shows that a large change in correlation is associated with a change in the effective rate of both oscillators, effected by a change in the intrinsic rate of one of them, and without a change in the connectivity.

A similar phenomenon can be observed in a simple two-spin Ising system, subject to an inhomogeneous local field (which represents the "intrinsic" activity). The Hamiltonian for this system is

$$H = -Js_1s_2 - h_1s_1 - h_2s_2 \tag{2}$$

where for simplicity we set $\beta = 1/kT = 1$. Thermodynamic averages can be derived from the partition function

$$\begin{aligned} Z &= \sum_{\{s_1,s_2\}} e^{-H} \\ \langle s_1s_2\rangle &= \frac{1}{Z}\frac{\partial Z}{\partial J} \\ \langle s_i\rangle &= \frac{1}{Z}\frac{\partial Z}{\partial h_i} \end{aligned} \tag{3}$$

where the different spin configurations can be trivially computed. Fixing the local field for one of the spins at $h_2 = 1$, we can compute the correlation and mean magnetization for both spins as a function of changes in J and $h_1 = h$, shown in Figure S2. As indicated by the arrows, it is possible to significantly change the correlation and the magnetization without a change in the coupling strength (black arrow), or change the coupling with little effect on the magnetization (red arrow).

This effect can also be observed in a linear system driven by uncorrelated noise. A system described by

$$\dot{\vec{x}} = A\vec{x} + Q\vec{\eta}(t) \tag{4}$$

where A represents the network connectivity (including self-connections A_{ii}) and Q the noise/input matrix, will reach steady-state (provided that $\forall i, \Re(\lambda_i) < 0$), characterized by the correlation matrix $C == C(0)$ and delayed correlations $C(\tau)$ satisfying

$$\begin{aligned} AC + CA^T &= -QQ^T \\ C(\tau) &= Ce^{\tau A^T} \end{aligned} \tag{5}$$

It is possible to affect the correlation matrix C without changes in A by simply changing the input rate. This can be seen in a simple example. Consider the "one link" system $x \rightarrow y$

$$\begin{aligned} \dot{x} &= -\mu x + a\eta_1(t) \\ \dot{y} &= -\mu y + bx + \eta_2(t) \end{aligned} \tag{6}$$

with solution

$$\begin{aligned} C_{11} &= \frac{a^2}{2\mu} \\ C_{12} &= \frac{ba^2}{4\mu^2} \\ C_{22} &= \frac{b^2a^2 + 2\mu^2}{4\mu^3} \end{aligned} \tag{7}$$

which shows that unless eq. 5 is fully resolved, including the input term and the possible non-symmetric nature of A, based solely on C_{12} one would conclude that the connectivity has changed.

Similarly, it is possible to change the connectivity without affecting the zero-lag correlation matrix. The formal solution to the Lyapunov equation is:

$$C = \int_0^\infty e^{At} QQ^T e^{A^T t} dt \tag{8}$$

Assuming a diagonal input, $QQ^T \sim I$, it can be readily seen that any antisymmetric change $\delta M = -\delta M^T$ will leave C unchanged. This would be the case, for instance, of reverting the directions of a feedback loop. Unless delayed correlation are computed, no differences would be observed. However, this change in structure without change in correlation can only be achieved in this very special condition.

In summary, we see that in widely different models of network interactions, a change in structure implies a change in correlations but not necessarily a change in the rate or mean activity of the units, while conversely a change in rate may change correlations without a change in the connectivity structure.

Classification Results with Unnormalized Activation Maps

We observed that normalization was essential for improving the performance of activation maps when using sparse MRF classifiers; while the raw activation maps perform similarly to normalized ones in case of GNB and SVM, their performance degrades dramatically (to 40-50% error) when using MRFs: (compare Figure S3c with the Figure 10c in the main paper).

Network Features: Detailed Classification Results

Figure S4 summarizes classification results on all network features, not included in the main text. While practically all network features attain a good accuracy of about 80% when using SVM on about 40,000 top-ranked features, pairwise correlations outperform all other feature types when using a smaller number of features. Particularly, the best accuracy of 93% is achieved using just 12 top-ranked pairwise correlations, which suggests that voxel-level pairwise correlations can be highly discriminative statistical biomarkers of schizophrenia.

Global Topological Features

Various *global* topological features were extracted: (1) the *mean degree*, i.e. the number of links for each node (corresponding to a voxel), averaged over the entire network; (2) the *mean geodesic distance*, i.e. the minimal number of links needed to reach any to from any other node, averaged over the entire network; (3) the *mean clustering*, i.e. the fraction of triangulations formed by a node with its first neighbors relative to all possible triangulations, averaged over the entire network; (4) the *giant component*, i.e. the size (number of nodes) of the largest connected sub-graph in the network; (5) the *giant component ratio*, i.e. the ratio of the giant component to the size of the network; (6) the *total number of links* in the network. The list of these global features, their mean values for each group, and the p-values for the comparison between the schizophrenic group vs. normal+alcoholic group are shown in Table S1.

Controls for Movement Artifacts

(Lack of) Movement Effect on Our Features

In order to estimate the possible incidence of movement artifacts in the classification results, we developed an approach that addresses the issue directly in the context of predictive modeling; specifically, we computed pairwise correlations between the movement parameters and each network feature, for all feature types listed in the paper (e.g., for degree features, we computed correlation between a motion parameter and degree of each voxel). To see whether those correlations were significant, we used the FDR test with significance level 0.05, on p-values corresponding to the correlations (computed in standard way using MATLAB corr function). Note that none of them passed the significance test; e.g., Figure S5 shows the results for the degree features and link weight (voxel-voxel correlation) features, where FDR threshold line is shown in red, and p-values corresponding to a network feature are shown in blue; x-axis correspond to the indexes of sorted p-values and y axis to their values. As we can see, p-values are too high to pass the FDR threshold (except for maybe 1 voxel in case of degree features on the left).

Classification Results with Alcoholic Group

We also considered the task of discriminating between the schizophrenic group versus the aggregate of the normal group and a second control group formed by patients suffering from alcoholism. The rational to include this additional group was to test for possible non-specific disruptions in the signal simply due to the brain being in a dysfunctional state. Moreover, a prominent feature of this group is a higher level of movement inside the scanner, prior to movement corrections; therefore, the alcoholic group also provides a test for potential movement confounds. Indeed, the classification results are such that both these hypotheses (i.e. non-specificity and movement artifacts) can be rejected.

Table S2 show the results of classification using global features described above in Table S1. Observe that the mean activation shows a good statistical power (low p-values in Table S1); however, when these

global features are fed into a classifier (Table S2), the corresponding results for mean activation are quite poor, whereas the topological features display much better accuracy.

Next, Figure S6 shows the results for voxel-level features. In these experiments were focused on the comparison with activation map 8 that demonstrated best predictive performance when using SVM on the full set of voxels; while full exploration of all activation maps in the low-voxel regime was not yet performed with inclusion of alcoholic group, it is more important to see that the degree maps again demonstrate similarly high predictive power to the one observed in our main study (schizophrenic vs normal groups). In summary:

- Results of applying Gaussian Naïve Bayes classification to the degree maps, compared with the activation map, are shown in Figure S6a. Full degree maps achieve an error rate of 12% around 300 voxels, whereas contrast maps reach 22% for 3000 voxels. Moreover, the difference in the error rate for small number of voxels is remarkable, as contrast maps perform nearly at random level, whereas the degree maps perform consistently below 20% error.

- Support Vector Machine classification results are shown in Figure S6b. In this case, the best error rate (10%) is achieved by inter-hemispheric maps with around 200 voxels, which is indeed the least for all maps. Contrast maps, on the other hand, stay above 30% error for most pre-selected voxel sizes, and reach 35% for the size corresponding to the least degree map error of 10%.

- Sparse Markov Random Field results are shown in Figure S6c. Given the computational demands of this approach, we only show results for up to 300 pre-selected voxels. For contrast maps the error rate is consistently above 30%, although the densest model (0.0001) is significantly better than the sparser ones, which provides more evidence that, even for contrast maps, synergetic interactions are relevant. The picture is even sharper for degree maps: these maps are very sensitive to the sparsity parameter, with sparse models averaging 30% error, and denser ones reaching stable error rates of 12% for 0.001. Taking together, the comparison between contrast maps and sparser degree maps on the one hand, and on the other the denser degree maps, indicates that the functional synergy captured by the correlation networks, and included in the MRF model, provide a very significant signal for the classification task. This effect supports our hypothesis that schizophrenia implies a functional network disruption which is not reducible to an activation disruption.
